# Supplementary material for: A smart multiantenna gene theranostic system based on the programmed assembly of hypoxia-related siRNAs
Source: Nat Commun. 2021 Jun 25;12:3953. doi: 10.1038/s41467-021-24191-9 (PMC8233311; doi:10.1038/s41467-021-24191-9)
Supplement: Supplementary file 1 — Supporting Information [file 41467_2021_24191_MOESM1_ESM.pdf]

Supplementary Information

A smart multiantenna gene theranostic system based on the programmed assembly of hypoxia-related siRNAs

Xue Gong,[1] *Haizhou Wang,*[2] *Ruomeng Li,*[1] *Kaiyue Tan,*[1] *Jie Wei,*[1] *Jing Wang,*[1] *Chen Hong,*[1] *Jinhua Shang,*[1] *Xiaoqing Liu,*[*1] *Jing Liu,*[2] *Fuan Wang**[,1]

[1] Key Laboratory of Analytical Chemistry for Biology and Medicine (Ministry of Education), College of Chemistry and Molecular Sciences, Wuhan University, Wuhan, 430072, P. R. China

[2] Department of Gastroenterology, Zhongnan Hospital of Wuhan University, Wuhan, 430072, P. R. China

[*] To whom correspondence should be addressed. E-mail: fuanwang@whu.edu.cn; xiaoqingliu@whu.edu.cn

Supplementary Table 1 Sequences of the oligonucleotides for bis-RNAi

Name	Sequence(5'-3')
miR-21	UAG CUU AUC AGA CUG AUG UUG A
**H$_1$**	TCA ACA TCA GTC TGA TAA GCTA TGG ATG TGA TAG CTT ATC AGA CTG ATT TCT CTC ATT TCC TCA TG
**H$_1$'**	TCA ACA TCA GTC TGA TAA GCTA TGG ATG TGA TAG CTT ATC AGA CTG ATT TCT CTC ATT TCC TCA TG-**BHQ1**
**H$_1$-sc**	TCA ACA TCA GTC TGA TAA GCTA TGG ATG TGA TAG CTT ATC AGA CTG ATT TTT TTT TTT TTT TTT TT
**H$_2$**	TCT TGC TCA GCT TGT CCC TGA TAG CTT ATC AGA CTC CCC GGT GAT AAG CTA TCA CAT CCA
**H$_2$-sc**	TTT TTT TTT TTT TTT TTC TGA TAG CTT ATC AGA CTC CCC GGT GAT AAG CTA TCA CAT CCA
**H$_2$'**	**ICG**-TCT TGC TCA GCT TGT CCC TGA TAG CTT ATC AGA CTC CCC GGT GAT AAG CTA TCA CAT CCA
**H$_3$**	CAT GAG GAA ATG AGA GAA ATC AGC CGG GGA GTC TGA TAA GCT ACC CTA CCC TAG CTT ATC AGA CT
**H$_3$-sc**	AAA AAA AAA AAA AAA AAA ATC AGC CGG GGA GTC TGA TAA GCT ACC CTA CCC TAG CTT ATC AGA CT
**H$_4$**	TAG CTT ATC AGA CTG ATG TTG AAG TCT GAT AAG CTA GGG TAG GGT CAG GGA CAA GCT GAG CAA GAT T
**H$_{4\text{-s}}$**	TAG CTT ATC AGA CTG ATG TTG AAG TCT GAT AAG CTA GGG TAG GGT CAG AAA AAA AAA AAA AAA AAA A
**HIF-1α-sense**	CAU GAG GAA AUG AGA GAA ATT
**HIF-1α-anti**	UUU CUC UCA UUU CCU CAU GTT
**twist-sense**	GGA CAA GCU GAG CAA GAU U
**twist-anti**	AAU CUU GCU CAG CUU GUC CUU
**scrambled-sense**	AAA AAA AAA AAA AAA AAA ATT
**scrambled-anti**	UUU UUU UUU UUU UUU UUU UTT
**HIF-1α-sense'**	Cy3-CAU GAG GAA AUG AGA GAA ATT
**HIF-1α-anti'**	UUU CUC UCA UUU CCU CAU GTT-**Cy5**
**twist-sense'**	Cy3-GGA CAA GCU GAG CAA GAT T
**twist-anti'**	AAU CUU GCU CAG CUU GUC CTT-**Cy5**
**anti-miR-21**	mUmCmA mAmCmA mUmCmA mGmUmC mUmGmA mUmAmA mGmCmU mA
**Let-7a**	UGA GGU AGU AGG UUG UAU AGU U
**Three-SM**	UAG ***A***UU A***G***C AGA ***U***UG AUG UUG A
**Two-SM**	UAG ***A***UU AUC AGA ***U***UG AUG UUG A
**One-SM**	UAG CUU AUC A***C***A CUG AUG UUG A

mN = 2'-O-Me RNA base

The bold italic nucleotides of SM indicates the mismatched sequence

Supplementary Table 2 primers for qRT-PCR

**Name**	**Sequence(5'-3')**
**HIF-1α**	Forward: TCACCACAGGACAGTACAGGATGC<br>Reverse: CCAGCAAAGTTAAAGCATCAGGTTCC
**twist**	Forward: GGAGTCCGCAGTCTTACGAG<br>Reverse: TCTGGAGGACCTGGTAGAGG
**PDCD4**	Forward: AAACTCATCCCGGGACTC<br>Reverse: ATCCACCTCCTCCACATC
**PTEN**	Forward: TCCTCAGTTTGTGGTCTG<br>Reverse: TCTGGTCCTGGTATGAAG
**Caspase-3**	Forward: TGTGAGGCGGTTGTGGAAGAGT<br>Reverse: AATGGGGGAAGAGGCAGGTGCA
**β-actin**	Forward: AGCCTCGCCTTTGCCGA<br>Reverse: CTGGTGCCTGGGGCG
**GAPDH**	Forward: TGTGATGGGTGTGAACCACG<br>Reverse: CAGTGAGCTTCCCGTTCAGC
**miR-21**	TAGCTTATCAGACTGATGTTGA

Supplementary Table 3. Summary of different nanocarriers for siRNA delivery

siRNA delivery Systems	Loading efficiency	Ref.
PLGA nanoparticles	50 pmol per mg of PLGA	1
Mesoporous silica-coated polypyrrole nanoparticles	500 fmol per µg of nanoparticles	2
Gold nanoclusters	226 µmol per g gold nanoclusters	3
Gold nanorods	70 pmol per µg of gold nanorods	4
EVs	1.3 pmol per µg of EVs	Present study

Free energies calculations (ΔG)

Free energy of RNA and DNA duplex were calculated using NUPACK at 37 °C with equimolar concentration (1 μM).

Free energy (ΔG) of designed RNA/DNA hybrids was calculated according to the following approximate equation:

$$\Delta G_{RNA/DNA\ hybrids} \approx \frac{\Delta G_{DNA/DNA\ hybrids} + \Delta G_{RNA/RNA\ hybrids}}{2} \quad (1)$$

where $\Delta G_{DNA/DNA\ hybrids}$ and $\Delta G_{RNA/RNA\ hybrids}$ correspond to the free energy calculated for a DNA hybrids duplex and RNA hybrids duplex with identical sequence, respectively.

Free energy of the initial state was calculated as follows:

$$\Delta G_{initial} = \Delta G_{(RNA/DNA)1} + \Delta G_{(RNA/DNA)2} + \Delta G_{(RNA/DNA)3} + \Delta G_{(RNA/DNA)4} \quad (2)$$

Free energy of the final state was calculated:

$$\Delta G_{final} = \Delta G_{bis\text{-}siRNAs} + \Delta G_{HCR\ product} \quad (3)$$

Free energy of the bis-siRNAs was calculated:

$$\Delta G_{bis\text{-}siRNAs} = \Delta G_{siRNA1} + \Delta G_{siRNA2} \quad (4)$$

The difference in free energies between final and initial states was calculated:

$$\Delta\Delta G = \Delta G_{final} - \Delta G_{initial} \quad (5)$$

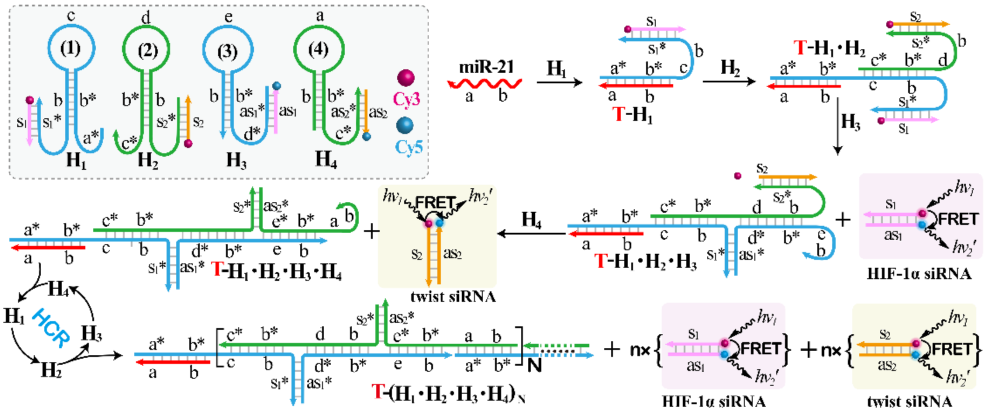

Supplementary Figure 1. Schematic illustration of the miR-21-activated HCR-amplified bis-RNAi operation.

The central idea is split the bis-siRNA into inactive sense and antisense RNAs. These single-strand RNAs were respectively hybridized with hairpin H_1, H_2, H_3 and H_4, resulting in the RNA/DNA hybrids assembly without their corresponding RNAi activity. The designed H_1 including the domain a*-b* that is complementary to the initiator miR-21. The initiator miR-21 opens H_1 by hybridizing with domain a*-b*, resulting in the formation of hybrid T-H_1. The as-exposed domain b-c of H_1 immediately hybridizes with domain c*-b* of H_2, yielding a structure T-$H_1 \cdot H_2$. The sequence d-b in the opened H_2 is exposed and successfully hybridizes with sequence d*-b* of H_3 with the formation of an intermediate T-$H_1 \cdot H_2 \cdot H_3$. The exposed single-stranded domain e-b of the opened H_3 hybridize with the sequence e*-b* of H_4 to construct an intermediate T-$H_3 \cdot H_4 \cdot H_5 \cdot H_6$ hybrid, giving rise to exposed single-stranded sequence a-b with an analog sequence of the target. Thus the exposed domain of a-b in the opened H_4 again opens H_1, leading to the continuous and successive cross-hybridization cycle. Simultaneously, the unlocked H_3 includes grafted sequence as_1* is bring close to H_1 that contains the corresponding complementary sequence s_1*, resulting in the proximity-induced mutual recognition for triggering toehold-mediated branch migration. This leads to the cyclic re-assembly of s_1/as_1 for efficiently activating the corresponding RNAi-1 system by HCR amplifier. At the same time, the unfolded H_2 and H_4 brought the separated segments s_2* and s_2* into close proximity, leading to the assembly siRNA-2. This brings the two fluorophores (Cy3 and Cy5) into close proximity and enables the Förster resonance energy transfer (FRET) readout.

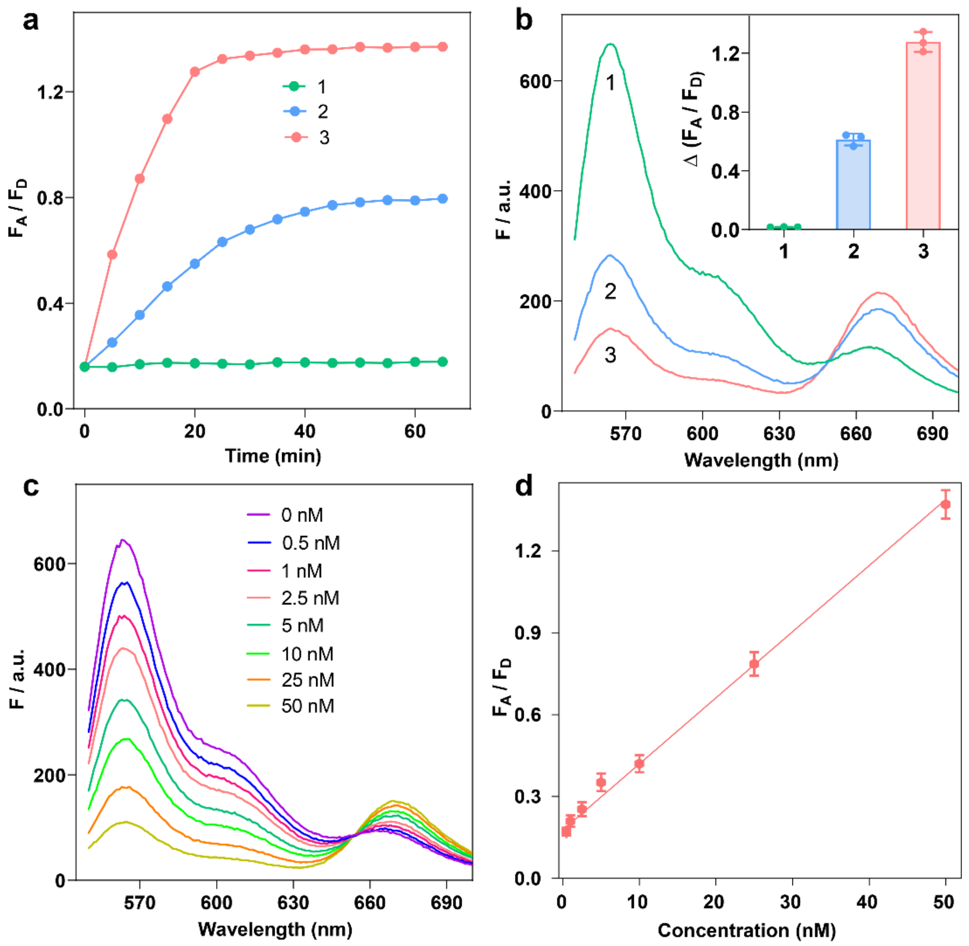

Supplementary Figure 2. **The feasibility and sensitivity of the miR-21 sensing platform. a**, Time-dependent fluorescence emission ratio of acceptor to donor (F_A/F_D) and (**b**) the corresponding fluorescence spectra of the HCR system after a fixed time-interval of 60 min in the absence of miR-21 (1), 35 nM miR-21 with one siRNA-released (2), 35 nM miR-21 with two siRNA-released (3). Inset: Summary of the fluorescence emission ratio changes. **c**, Fluorescence spectra generated by the two-siRNA released HCR system upon analyzing different concentration of miR-21. **d**, The corresponding calibration curve of Supplementary Figure 2c with a sensitivity corresponding to 1×10^{-10} M. a.u. represents arbitrary unit. Data represent the mean ± s.d. from three (**b**, **d**) independent experiments. The results (**a**, **c**) are representative of three independent experiments. The system consisting of 100 nM RNA/DNA hybrids was reacted at room temperature in reaction buffer (10 mM HEPES, 1 M NaCl, 50 mM $MgCl_2$, pH 7.2) for 60 min to acquire the fluorescence spectra.

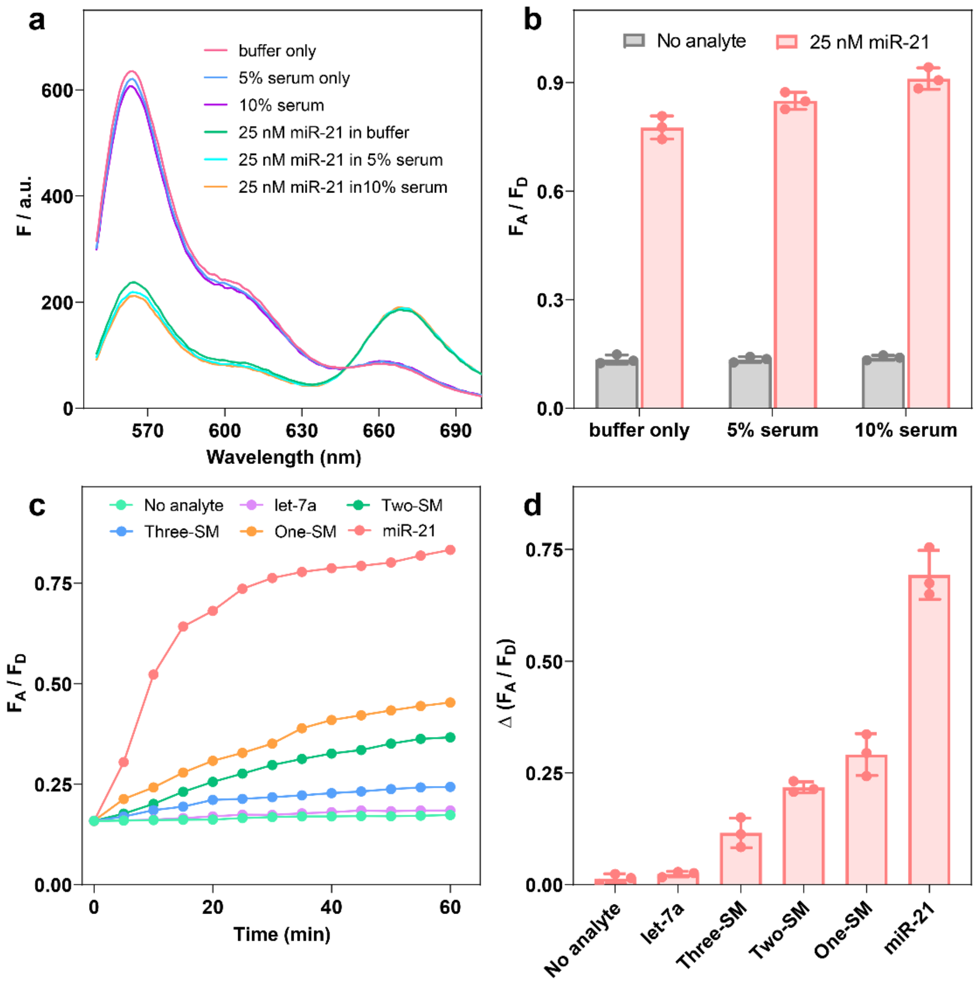

Supplementary Figure 3. The selectivity and stability of the RNAi prodrugs system. **a**, Fluorescence spectra generated of the HCR system upon analyzing miR-21 in different solutions. **b**, Summary of the fluorescence emission ratio of acceptor to donor (F_A/F_D) as shown in Supplementary Figure 3a. **c**, Time dependent fluorescence emission ratio of acceptor to donor (F_A/F_D) upon analysis of different analytes: no analyte, 25 nM let-7a, 25 nM Three SM, 25 nM Two-SM, 25 nM One-SM, 25 nM miR-21. **d**, Fluorescence emission ratio changes for different analytes after a fixed time-interval of 60 min. a.u. represents arbitrary unit. Data represent the mean ± s.d. from three (**b**, **d**) independent experiments. The system consisting of 100 nM RNA/DNA hybrids was reacted at room temperature in reaction buffer (10 mM HEPES, 1 M NaCl, 50 mM $MgCl_2$, pH 7.2) for 60 min to acquire the fluorescence spectra.

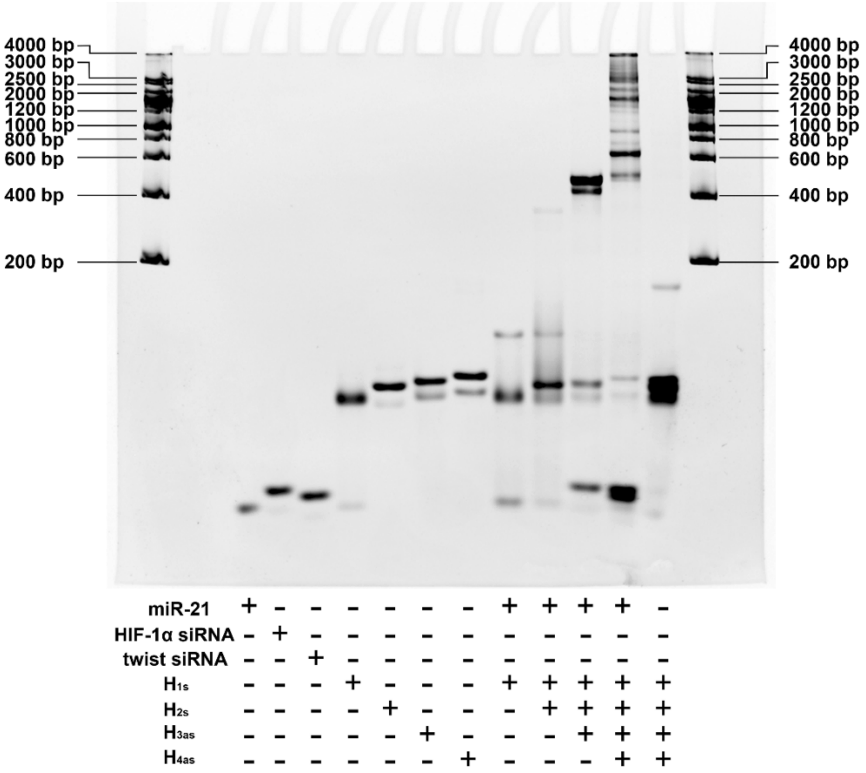

Supplementary Figure 4. **PAGE analysis of miR-21-activated HCR-promoted bis-siRNAs.** The concentration of all the RNA/DNA hybrids was fixed at 200 nM. The symbols “+” and “□”represent the presence and absence of relevant components, respectively. The gel images are representative of three independent experiments.

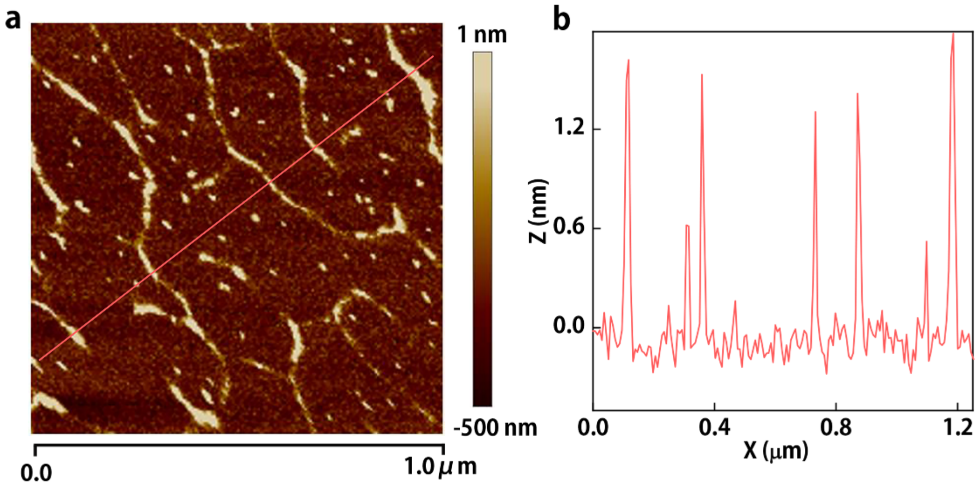

Supplementary Figure 5. Characterization of the dsDNA products. a, AFM characterization of miR-21-initiated HCR and (b) corresponding cross-sectional analysis. The AFM images are representative of three independent experiments.

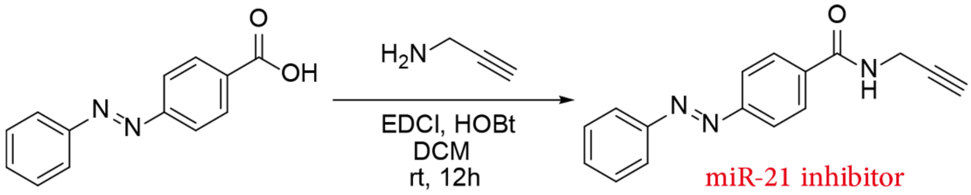

Supplementary Figure 6. Synthesis of the miR-21 inhibitor. 4-Phenylazobenzoic acid (30 mg, 0.133 mmol) was dissolved in DCM (1 mL), followed by the addition of 1-ethyl-3-(3'-dimethylaminopropyl) carbodiimide (42 mg, 0.22 mmol) and hydroxybenzotriazole (21 mg, 0.15 mmol). Propargylamine (15 mg, 0.27 mmol) was added, and the mixture was stirring for 12 h at room temperature. The reaction was quenched with water (5mL) and extracted with DCM (3 X 5mL). The organic layer was dried with sodium sulfate, concentrated and purified by silica gel chromatography (2:1 hexane/ethyl acetate) to yield an orange solid (27 mg, 0.10 mmol, 75%).

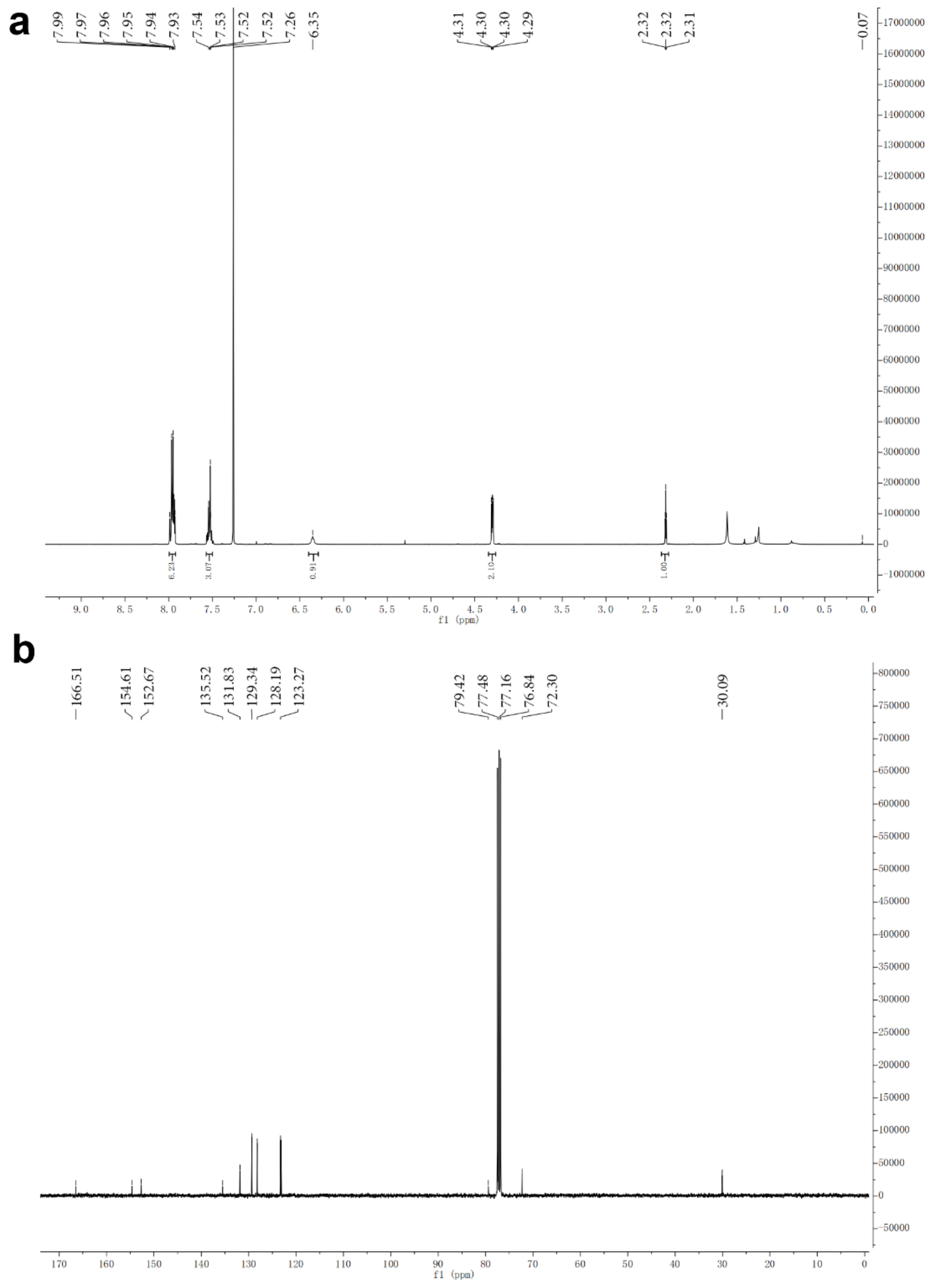

Supplementary Figure 7. Characterization of the miR-21 inhibitor. a, ^{1}H NMR spectra of synthesized miR-21 inhibitor in $CDCl_3$. ^{1}H NMR (400 MHz, $CDCl_3$): δ 7.99-7.93 (m, 6H), 7.54-7.52 (m, 3H), 6.35 (s, 1H), 4.30 (dd, J = 4 Hz, 2H), 2.32 (t, 1H). **b**, ^{13}C NMR spectra of miR-21 inhibitor in $CDCl_3$. ^{13}C NMR (100 MHz, $CDCl_3$): δ 166.51, 154.61, 152.67, 135.52, 131.83, 129.34, 128.19, 123.27, 79.42, 72.30, 30.09.

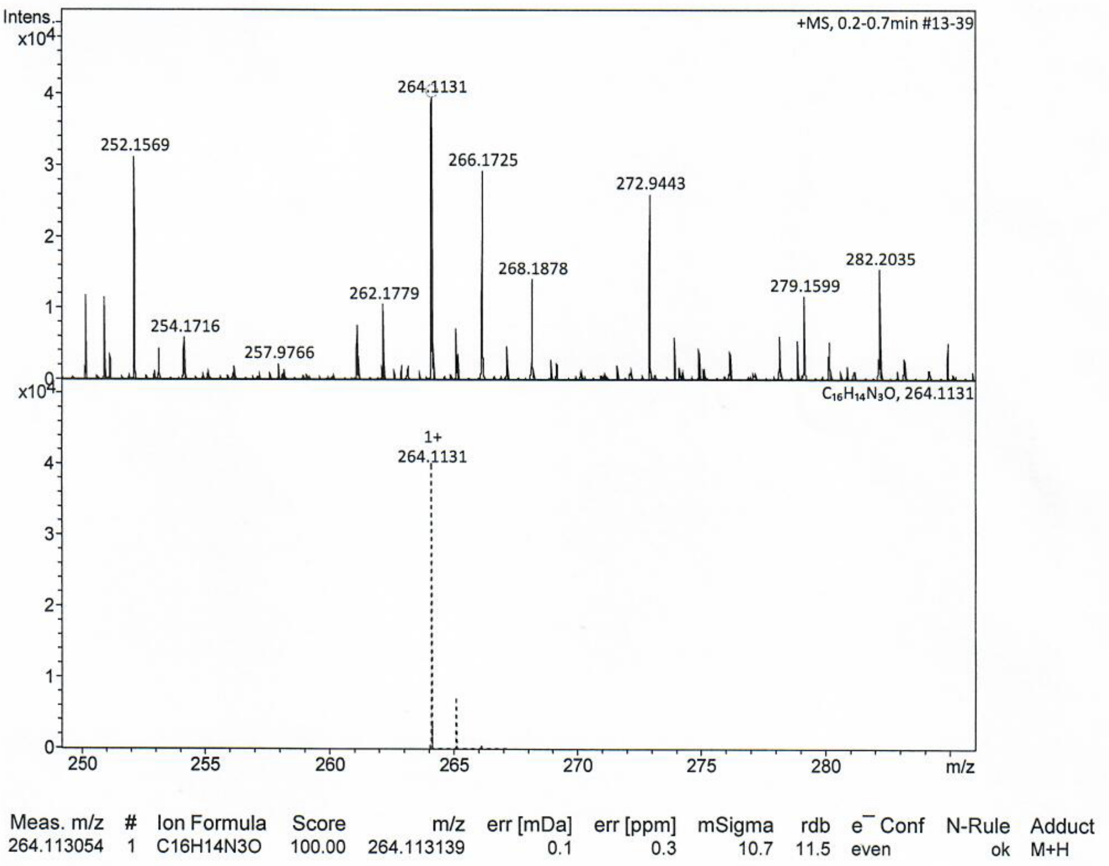

Supplementary Figure 8. **The HRMS spectrum of miR-21 inhibitor compound**. HRMS (ESI): m/z calculated for $C_{16}H_{14}N_3O$ $[M+H]^+$ 264.1131, found 264.1131.

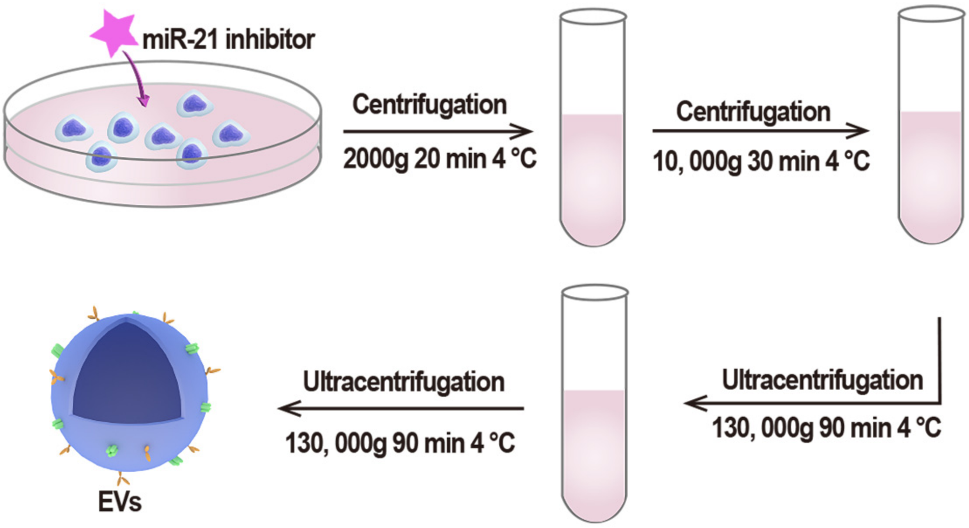

Supplementary Figure 9. Purification protocol of EVs derived from MDA-MB-231 cells.

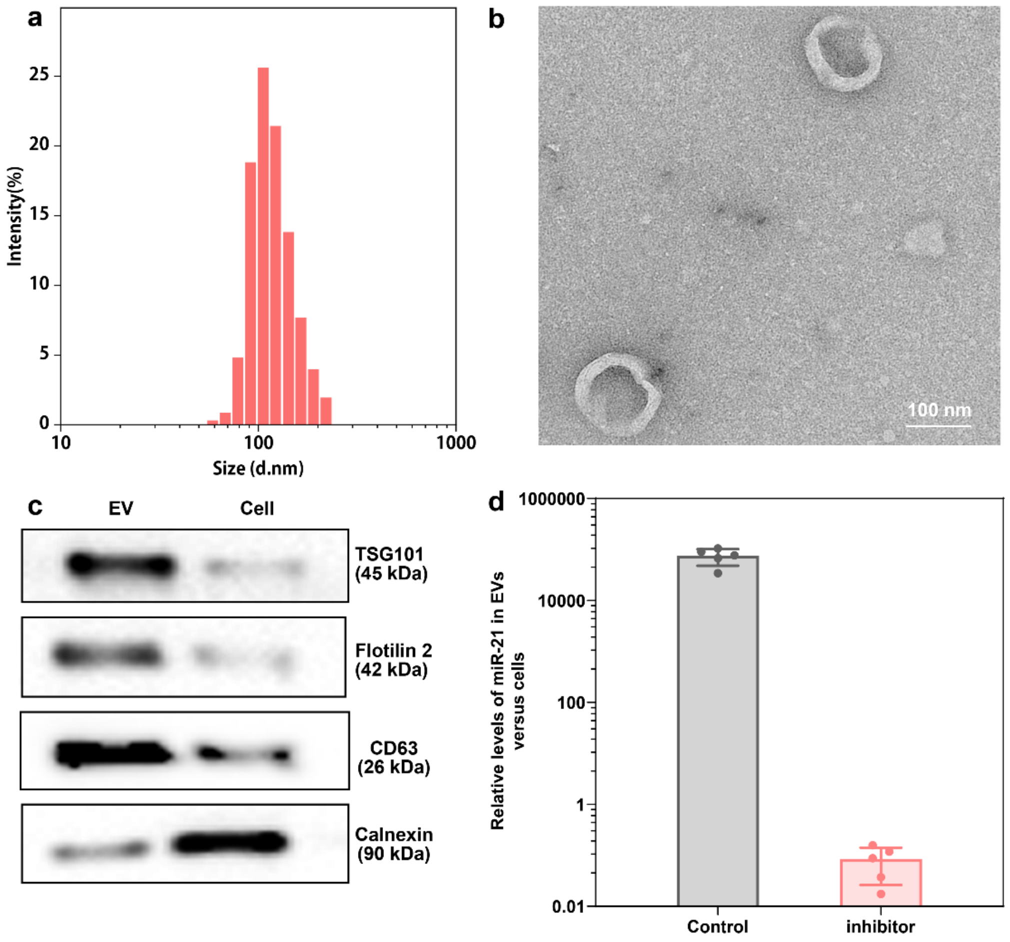

Supplementary Figure 10. Characterization of the isolated EVs derived from MDA-MB-231 cells. a, Size distribution of EVs. **b**, TEM characterization of EVs. **c**, Western blot analysis of EVs and the corresponding cell lysates. **d**, qRT-PCR analysis the content of miR-21 in EVs vs. its expression in MDA-MB-231 cells with miR-21-inhibitor pre-treatment (normalized to U6 RNA). Data represent the mean ± s.d. from five (**d**) independent replicates. The characteristic results (DLS, TEM, western blot image) representative of three independent experiments.

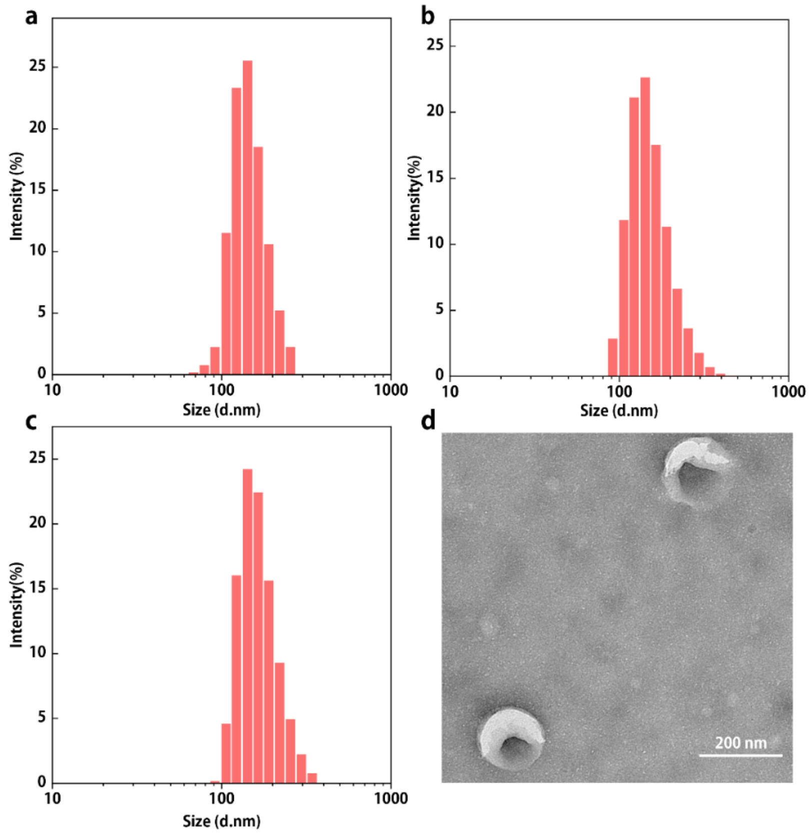

Supplementary Figure 11. Characterization of EVs that were treated with multiple freeze-thaw cycles. Size distribution of EVs after one freeze-thaw cycle (**a**), two freeze-thaw cycles (**b**) and three freeze-thaw cycles (**c**). **d**, Representative TEM image of EVs after three freeze-thaw cycles. The characteristic results (DLS, TEM) representative of three independent experiments.

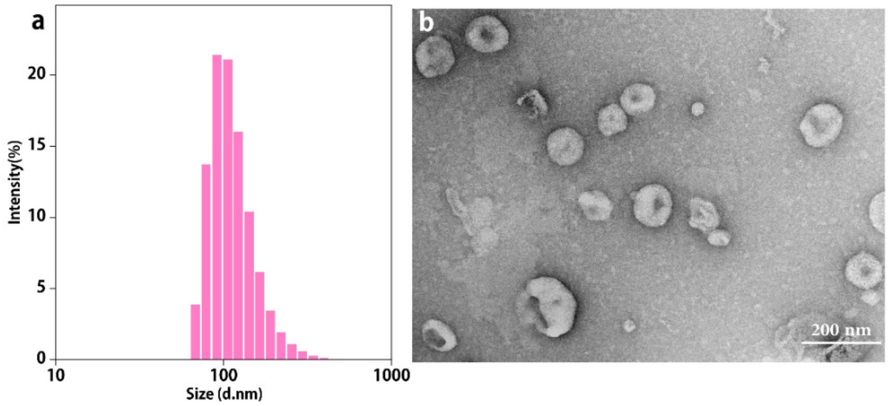

Supplementary Figure 12. Characterization of RNAi prodrugs. a, Size distribution of RNAi prodrug aggregates after electroporation as measured by DLS. **b**, Representative TEM image of RNAi prodrugs-packaged EVs. The size distribution histogram and TEM images are representative of three independent experiments.

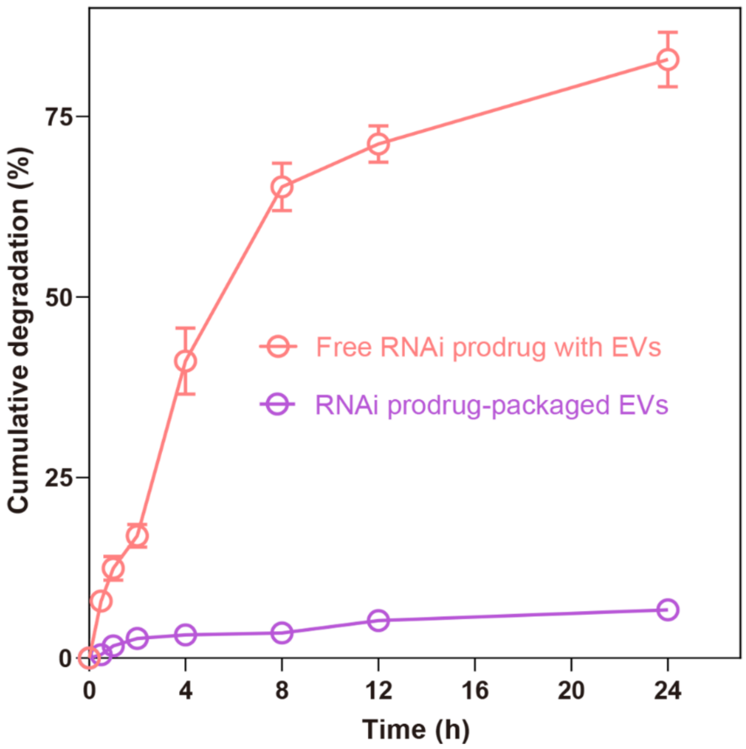

Supplementary Figure 13. The RNAi prodrugs degradation profile in Opti-MEM containing 10 U RNase H and 10 U DNase at 37 °C. Data represent the mean ± s.d. of three independent experiments. Here, H_1 is functionalized at its 3'-end with BHQ1 while s_1 is modified at its 5'-end with Cy3.

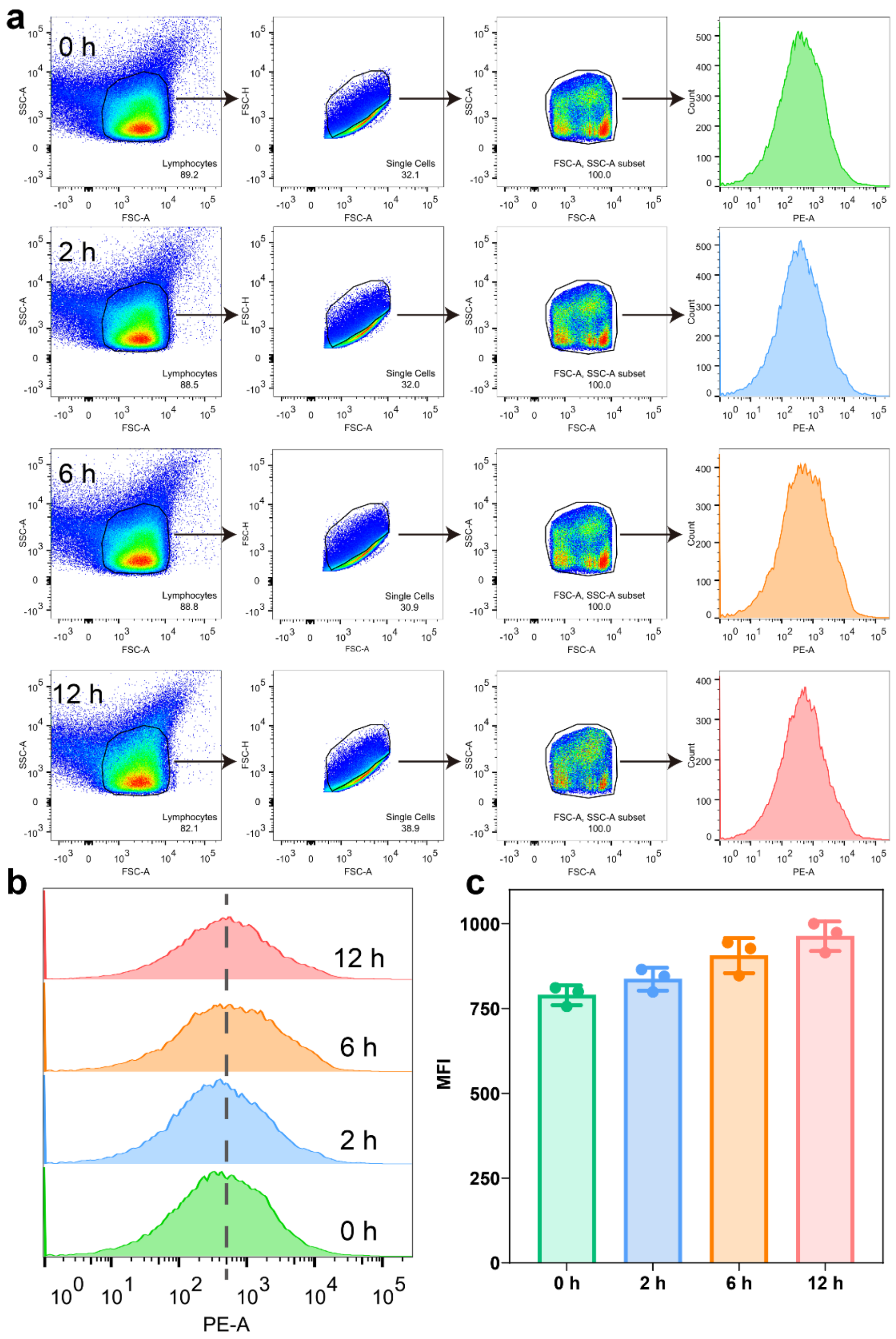

Supplementary Figure 14. Stability of RNAi prodrugs in EVs. (a, b) Flow cytometric analysis and (**c**) the corresponding mean fluorescence intensity (MFI) of RNAi prodrugs in EVs. Data represent the mean ± s.d. of three (**c**) independent replicates. Here, H_1 is functionalized at its 3’-end with BHQ1 while s_1 is modified at its 5’-end with Cy3.

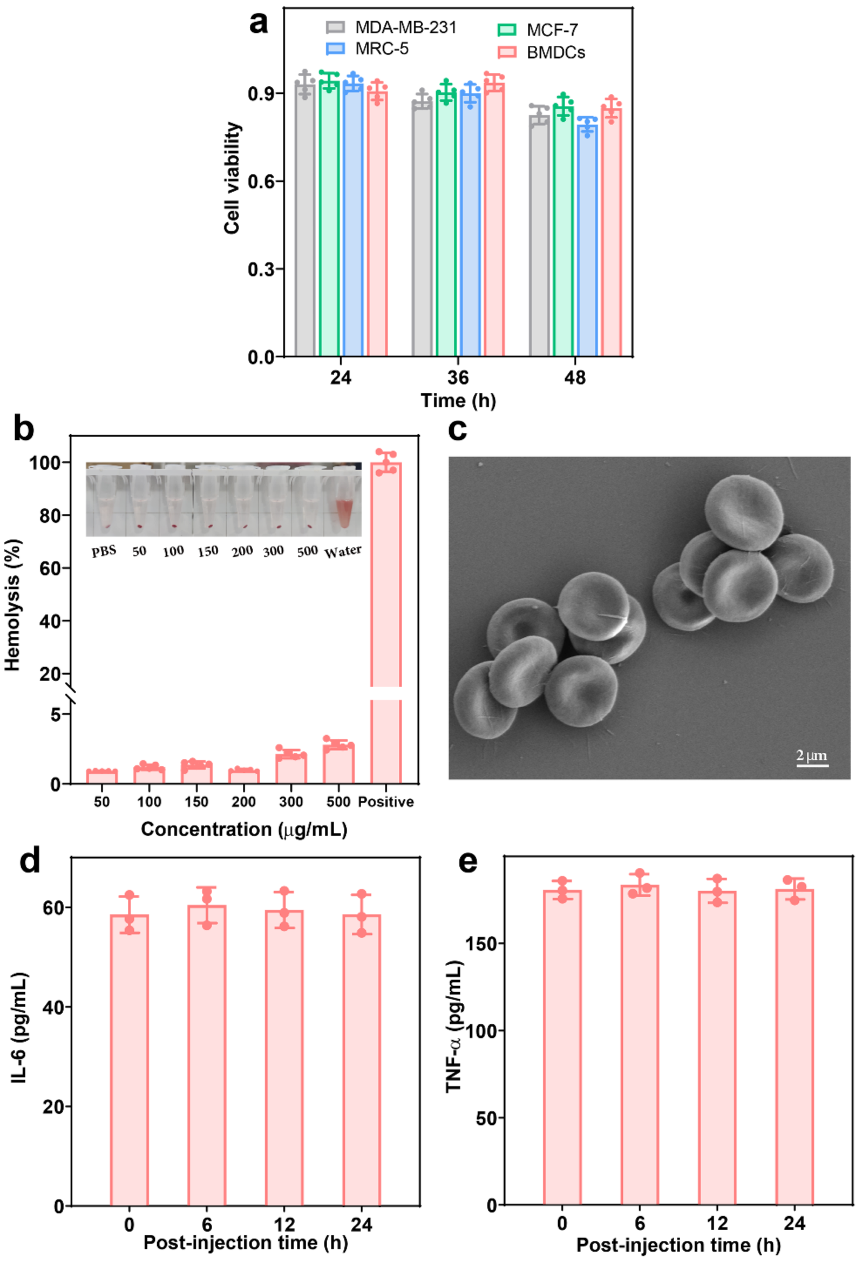

Supplementary Figure 15. **The biocompatibility evaluation of EVs. a**, Cell viability assay of MDA-MB-231, HeLa, MRC-5 and BMDCs cells after their incubation with bare EVs (225 μg/mL) for varied durations. **b**, Hemolytic analysis of RNAi prodrugs-packaged EVs at varied concentrations. PBS and water were used as negative and positive control, respectively. **c**, SEM image of RBCs after its treatment with RNAi prodrugs-loaded EVs (500 μg/mL) for 6 h. The respective expression level of IL-6 (**d**) and TNF-α (**e**) as measured by ELISA kit after the C57BL/6 mice was administrated with bare EVs for different durations. Data represent the mean ± s.d. of either five (**a**, **c**) or three (**d**, **e**) independent replicates. SEM image represent three independent experiments.

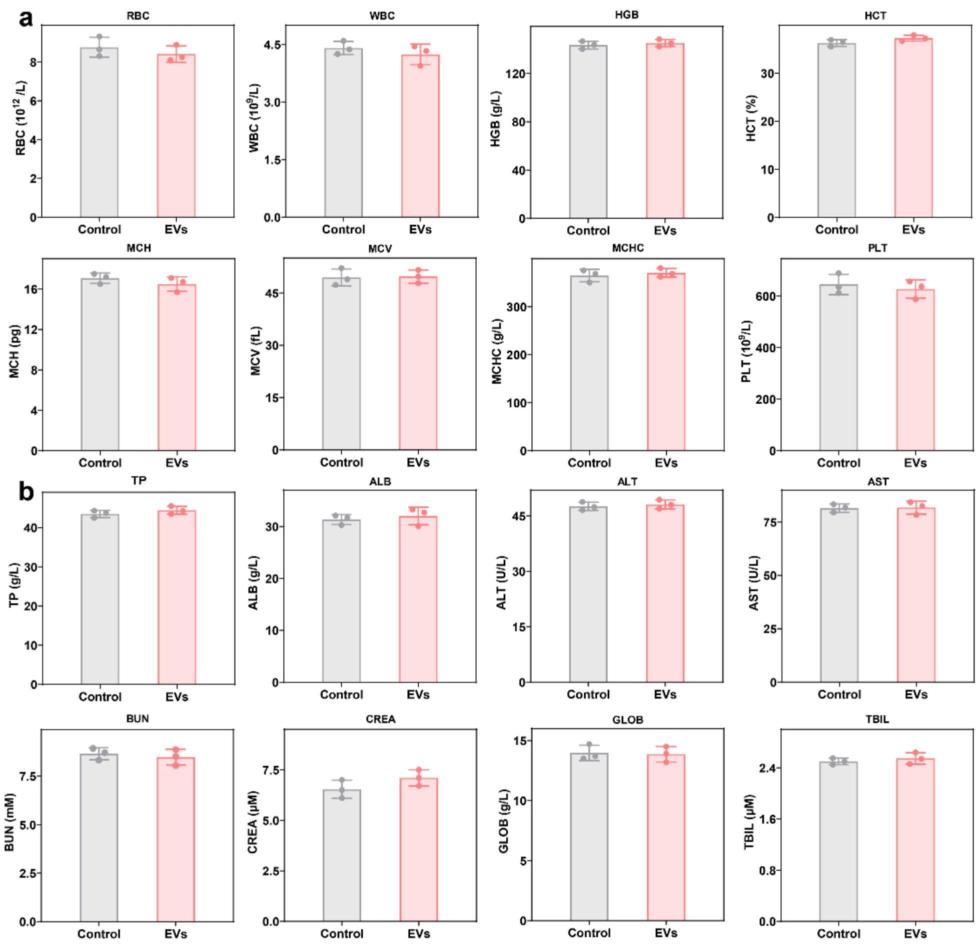

Supplementary Figure 16. **Biosafety evaluation of the EVs nanovesicles in vivo. a**, Whole blood cell analysis of mice after 6 weeks post intravenous injection bare EVs. RBC, red blood cell; WBC, white blood cell; HGB, hemoglobin; HCT, hematocrit; MCH, mean corpuscular hemoglobin; MCV, mean corpuscular volume; MCHC, mean corpusular hemoglobin concentration, PLT, platelet count. **b**, Hepatic and renal functions analysis of the intravenously injected mice with bare EVs after 6 weeks. TP, total protein; ALB, albumin; ALT, alanine aminotransferase; AST, aspartate aminotransferase; BUN, blood urea nitrogen; CREA, creatinine; GLOB, globulin; TBIL, total bilirubin. Data represent the mean ± s.d. of three (**a**, **b**) independent replicates.

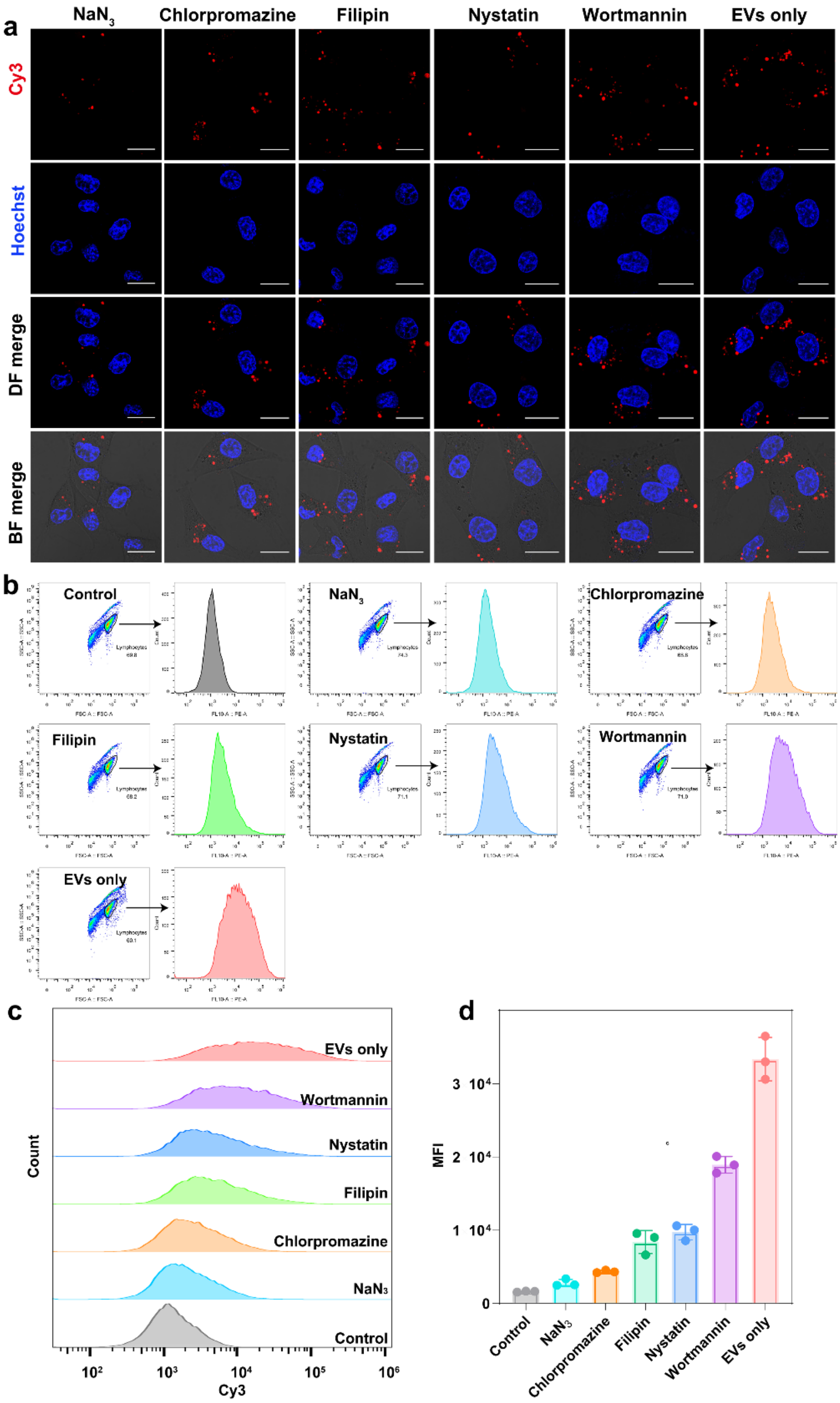

Supplementary Figure 17. The exploration of the uptake mechanism of RNAi prodrugs-packaged EVs. Confocal microscopy images (**a**) and quantification by analytical flow cytometry (**b**, **c**, **d**) of the internalized RNAi prodrugs in the presence of several inhibitors. All scale bars are 20 µm. Data represent the mean ± s.d. of three (**d**) independent replicates. The confocal micrographs are representative of three independent experiments.

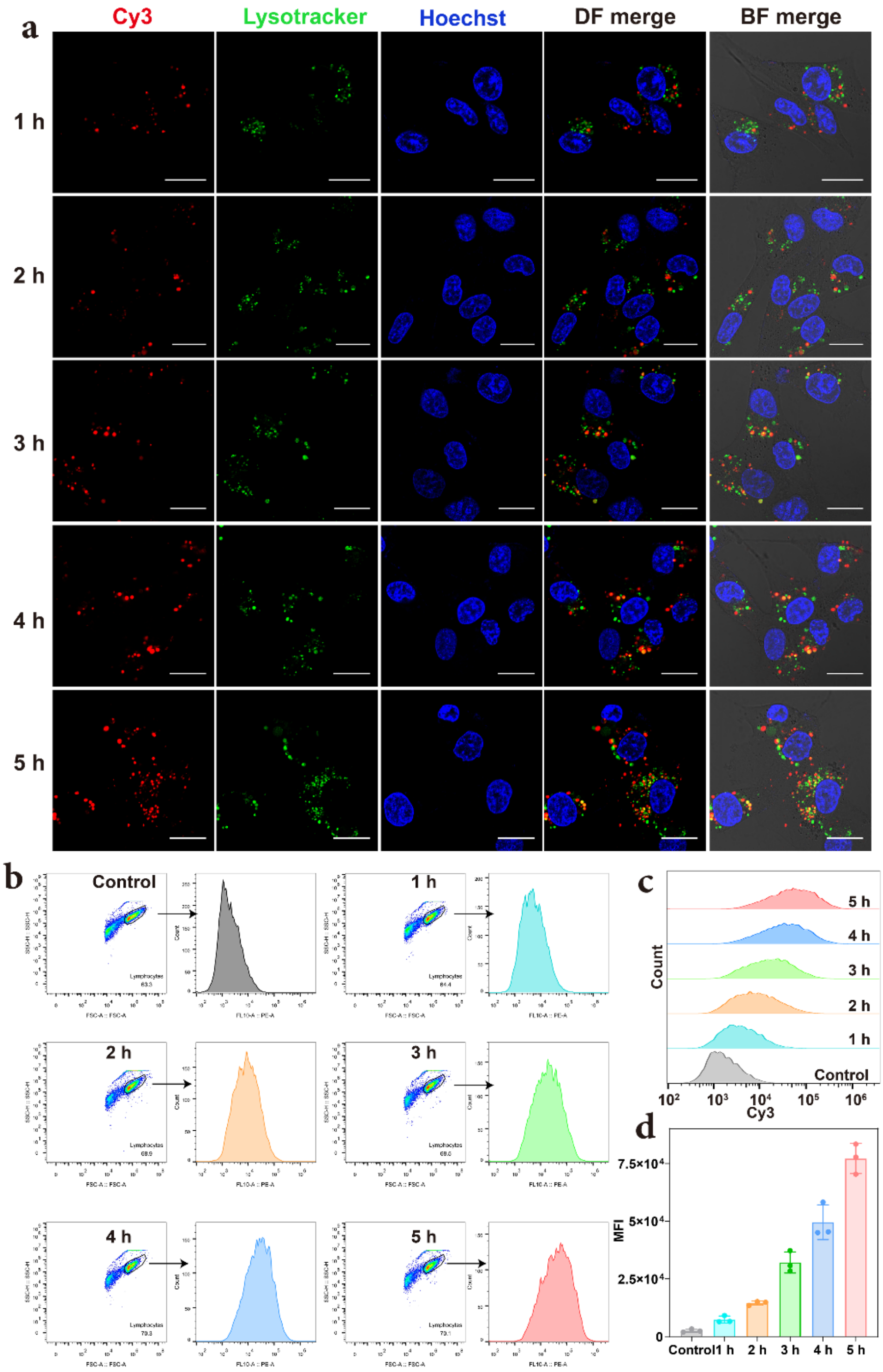

Supplementary Figure 18. Uptake of RNAi prodrugs by MDA-MB-231 cells over time. a, Lysosomal colocalization in cells incubated RNAi prodrug-packaged EVs for varied durations. All scale bars are 20 μm. **b**, Flow cytometric analysis and (**c**) the corresponding mean fluorescence intensity (MFI) in cells incubated with RNAi prodrug-packaged EVs for varied durations. Data represent the mean ± s.d. of three (**c**) independent replicates. The confocal micrographs are representative of three independent experiments.

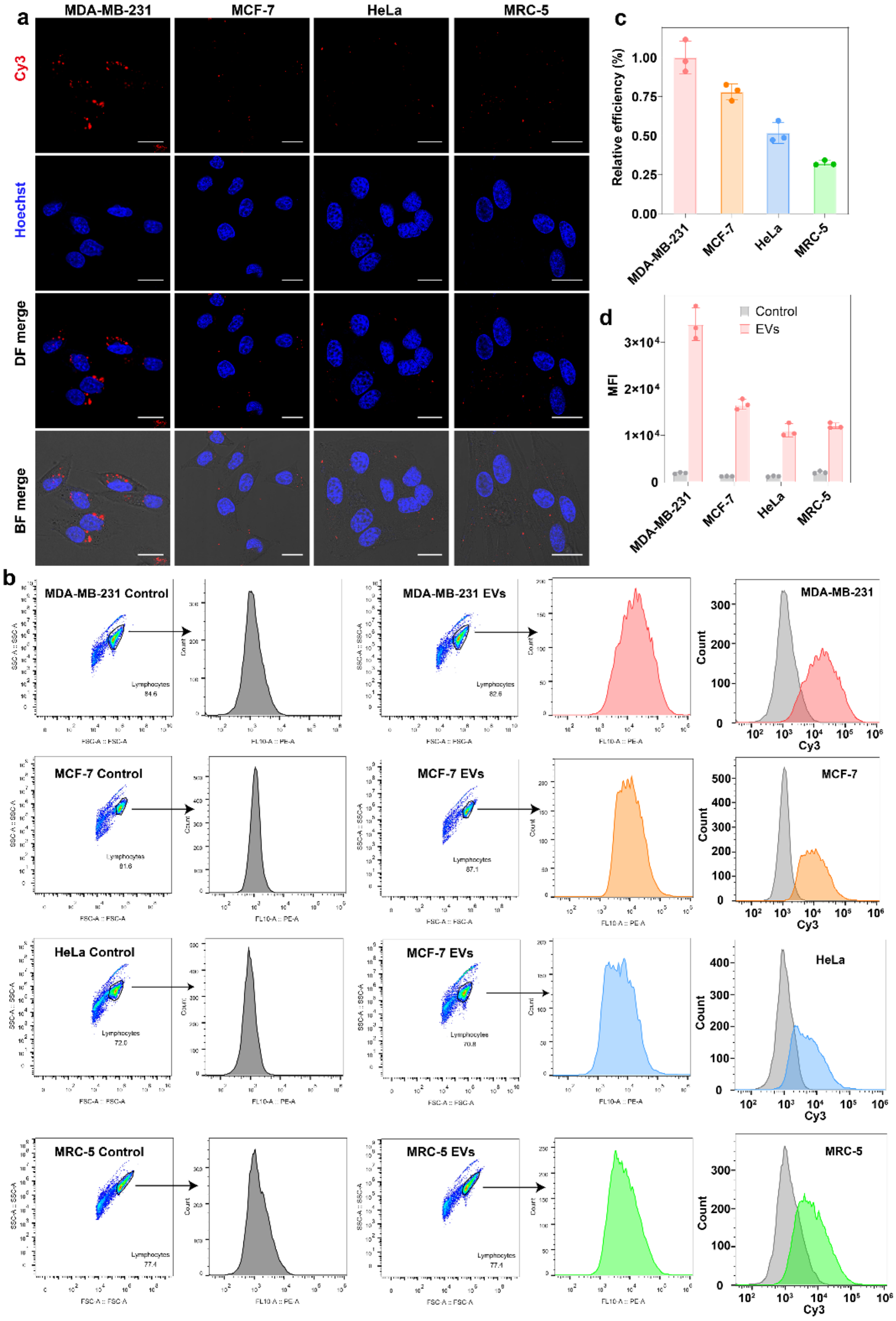

Supplementary Figure 19. **Homotypic targeting evaluation of EVs. a**, CLSM image of MDA-MB-231, HeLa, MCF-7 and MRC-5 cells that were incubated with RNAi prodrugs-loaded EVs derived from MDA-MB-231 cells for 3 h. The cells nuclei were stained with Hoechst 33342 (blue). All scale bars are 20 μm. **b**, Flow cytometry analysis of the RNAi prodrugs in different cells. **c**, Quantitative analysis of the intracellular fluorescence intensity of RNAi prodrugs in different cells. **d**, The relative uptake efficiency of different cells as compared to the MDA-MB-231 cells. Data represent the mean ± s.d. of three (**c**, **d**) independent replicates. The confocal micrographs are representative of three independent experiments.

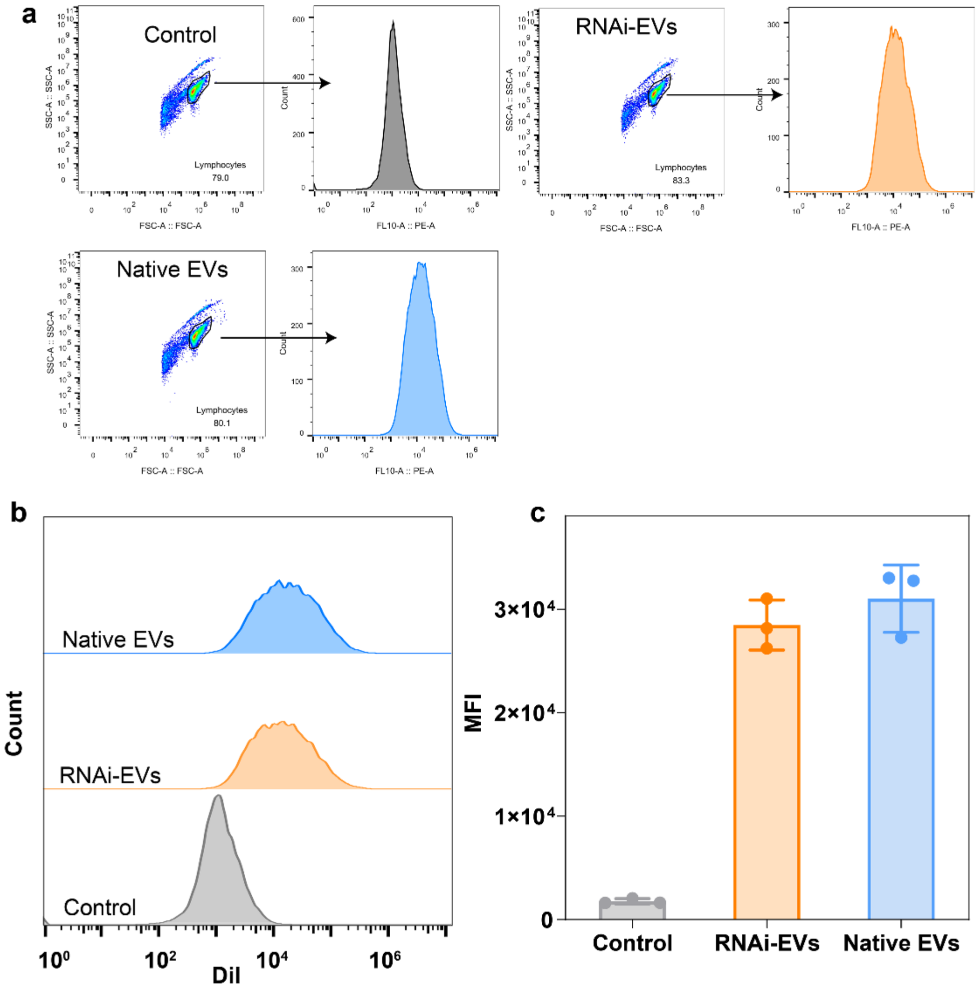

Supplementary Figure 20. Demonstration of the EVs uptake by MDA-MB-231 cells. **a**, Flow cytometry analysis and (**b**) the corresponding mean fluorescence intensity (MFI) of MDA-MB-231 cells that were incubated with RNAi prodrugs-electroporated DiI-labeled EVs or DiI-labeled bare EVs for 3 h. Data represent the mean ± s.d. of three (**b**) independent replicates.

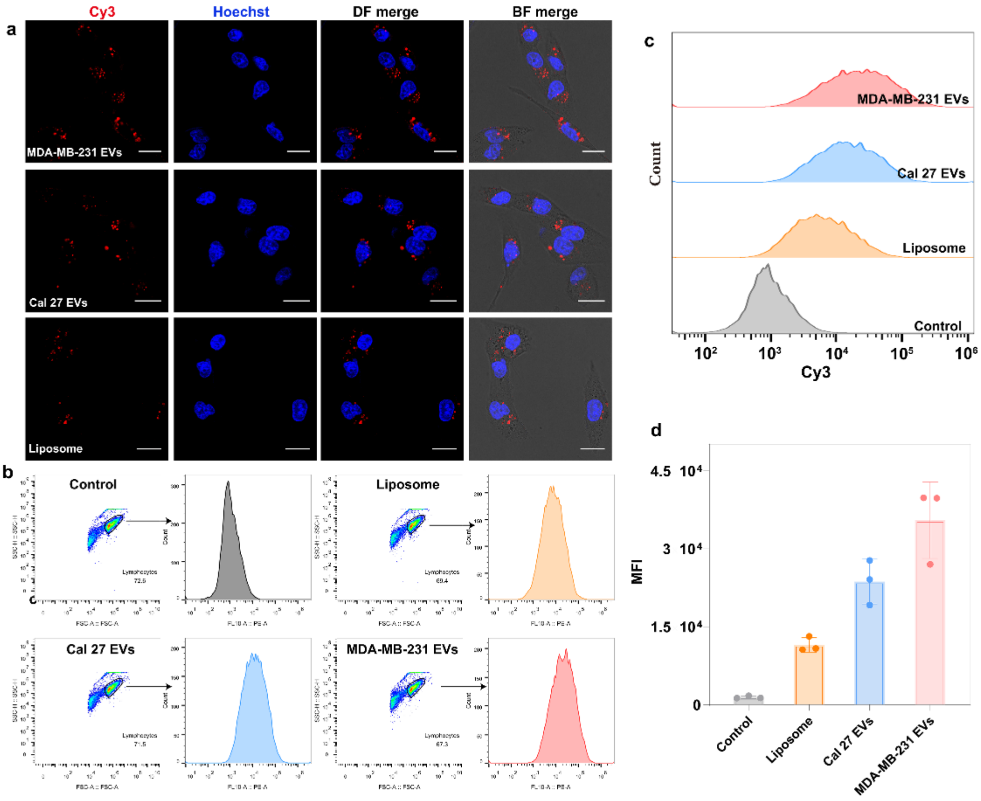

Supplementary Figure 21. Demonstration of the homotypic affinity of EVs towards donor tumor cells. a, CLSM images of MDA-MB-231 cells incubated with MDA-MB-231-derived EVs, Cal 27-derived EVs and liposome that were transfected with an equivalent amount of Cy3-labeled RNAi prodrugs for 3 h. All scale bars are 20 μm. **b**, Flow cytometric analysis and (**c**) the corresponding mean fluorescence intensity (MFI) of MDA-MB-231 cells incubated with MDA-MB-231-derived EVs, Cal 27-derived EVs and liposome that were transfected with an equivalent amount of Cy3-labeled RNAi prodrugs for 3 h. Data represent the mean ± s.d. of three (**c**) independent replicates. The confocal micrographs are representative of three independent experiments.

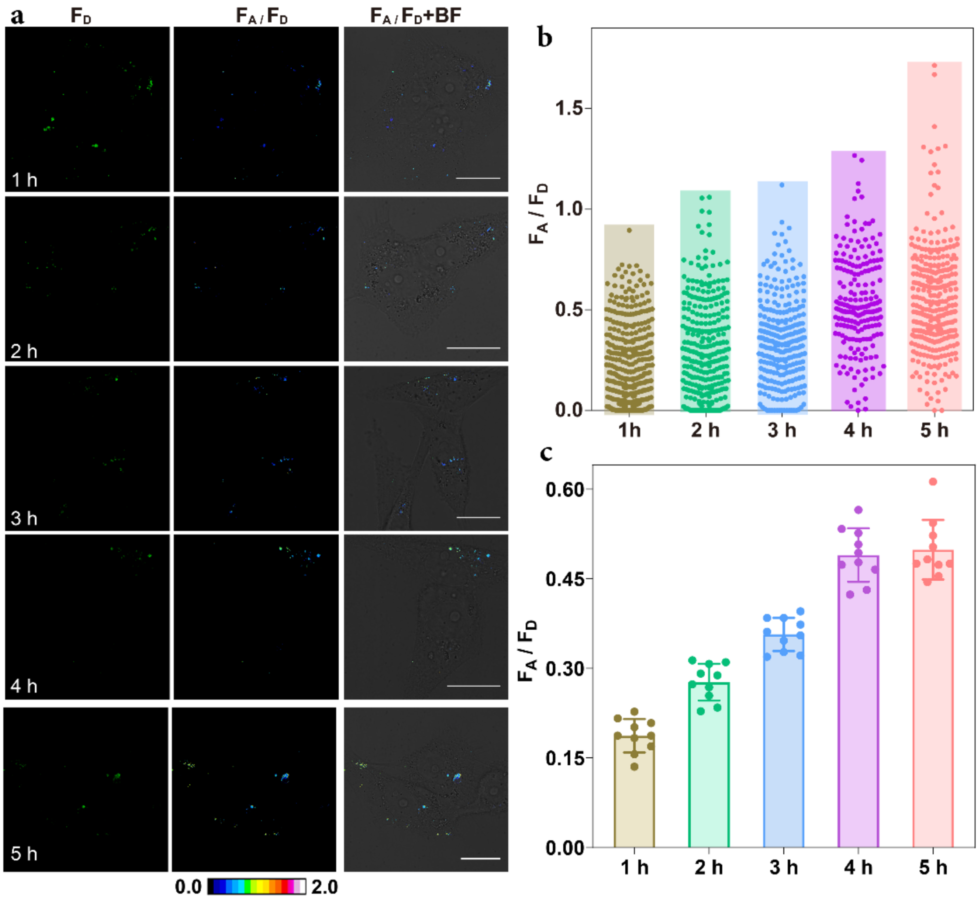

Supplementary Figure 22. Time-dependent miR-21 imaging in MDA-MB-231 cells. a, CLSM analysis of the MDA-MB-231 cells incubated with the RNAi prodrug-packaged EVs system for different time-intervals. The images are representative of ten independent experiments. All scale bars are 20 μm. **b**, FRET signal distributions of each pixel in Supplementary Figure 22a. **c**, Statistical histogram analysis of the relative fluorescence intensity (in the form of F_A/F_D) of the above five cell samples. Data represent the mean ± s.d. of ten (c) independent experiments.

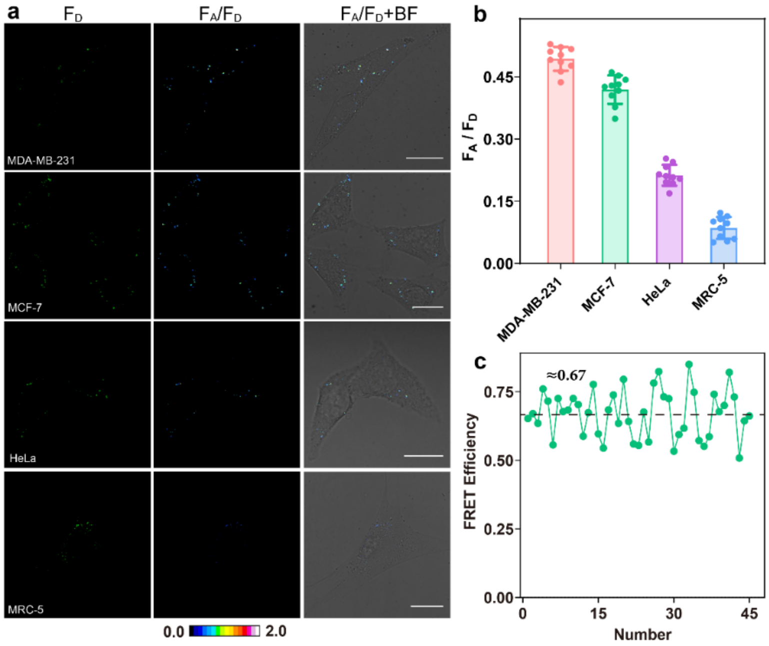

Supplementary Figure 23. **CLSM analysis of endogenous miR-21 by the RNAi prodrug-packaged EVs system. a**, Diagnostic EV-sustained HCR-amplified FRET analysis of intracellular miR-21 (in the form of F_A/F_D) in different cell lines. The images are representative of ten independent experiments. All scale bars are 20 μm. **b,** Statistical histogram analysis of the relative fluorescence intensity (in the form of F_A/F_D) of the above four cell samples. **c**, Determination of the FRET efficiency of HCR-imaging system in MDA-MB-231 cells. Data represent the mean ± s.d. of ten (**b**) independent experiments.

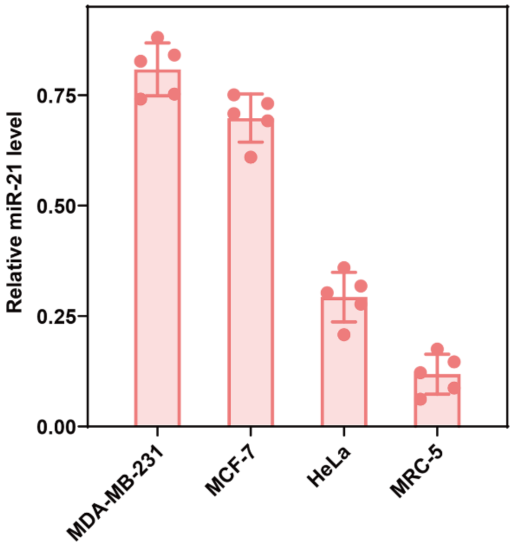

Supplementary Figure 24. The relative expressions of miR-21 in MDA-MB-231, MCF-7, HeLa and MRC-5 cells by using qRT-PCR evaluation. Data represent the mean ± s.d. of five independent replicates.

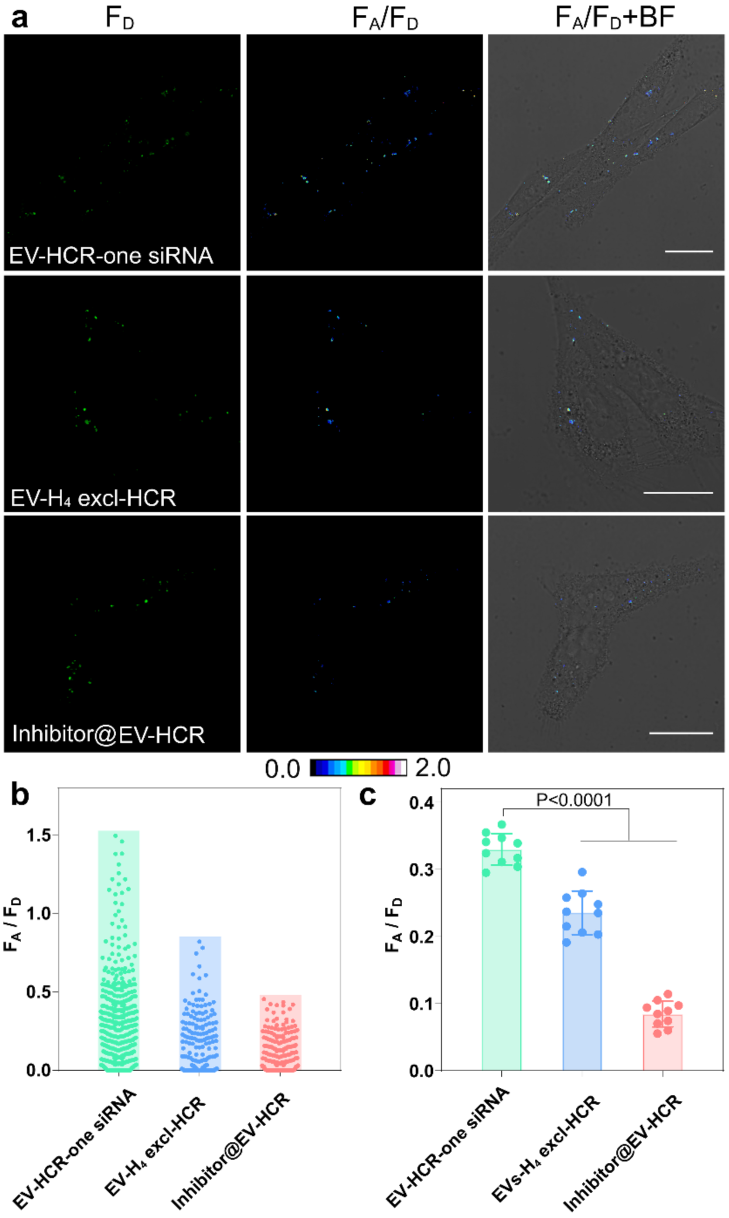

Supplementary Figure 25. **FRET analysis of miR-21 in living MDA-MB-231 cells based on different imaging system and FRET transduction (in the form of F_A/F_D).** **a,** CLSM imaging of miR-21 in MDA-MB-231 cells that were respectively incubated with the one-RNAi-contained HCR system, the H_4-excluded HCR system, and the miR-21 inhibitor pretreated HCR system. All scale bars are 20 μm. **b,** FRET signal distributions of each pixel in Supplementary Figure 25a. **c,** Statistical histogram analysis of the relative fluorescence intensity (in the form of F_A/F_D) of the above three cell samples. Data represent the mean ± s.d. of ten (**c**) independent measurements. The images are representative of ten independent experiments. Statistical significance is calculated by one-way ANOVA followed by Tukey's post hoc test.

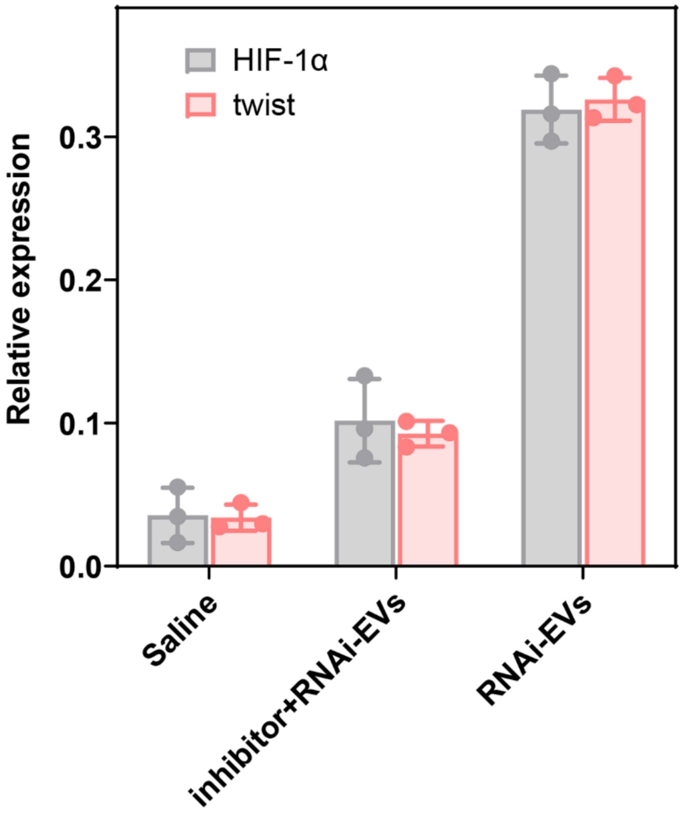

Supplementary Figure 26. qRT-PCR quantification of the relative expression of HIF-1α and twist siRNAs after different treatments. Data represent the mean ± s.d. of five independent replicates.

ESI-MS (m/z): $[ICG\text{-}H_2]^+$ calcd for 19164, found 19167.3.

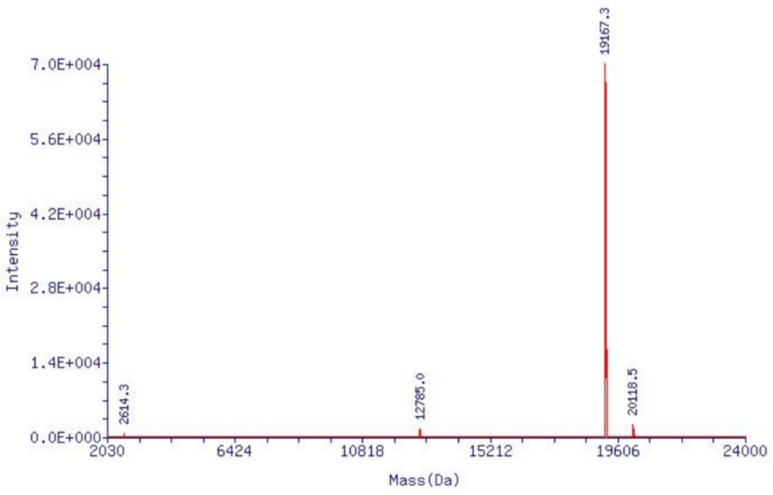

Supplementary Figure 27. ESI-MS analysis of ICG-functionalized DNA.

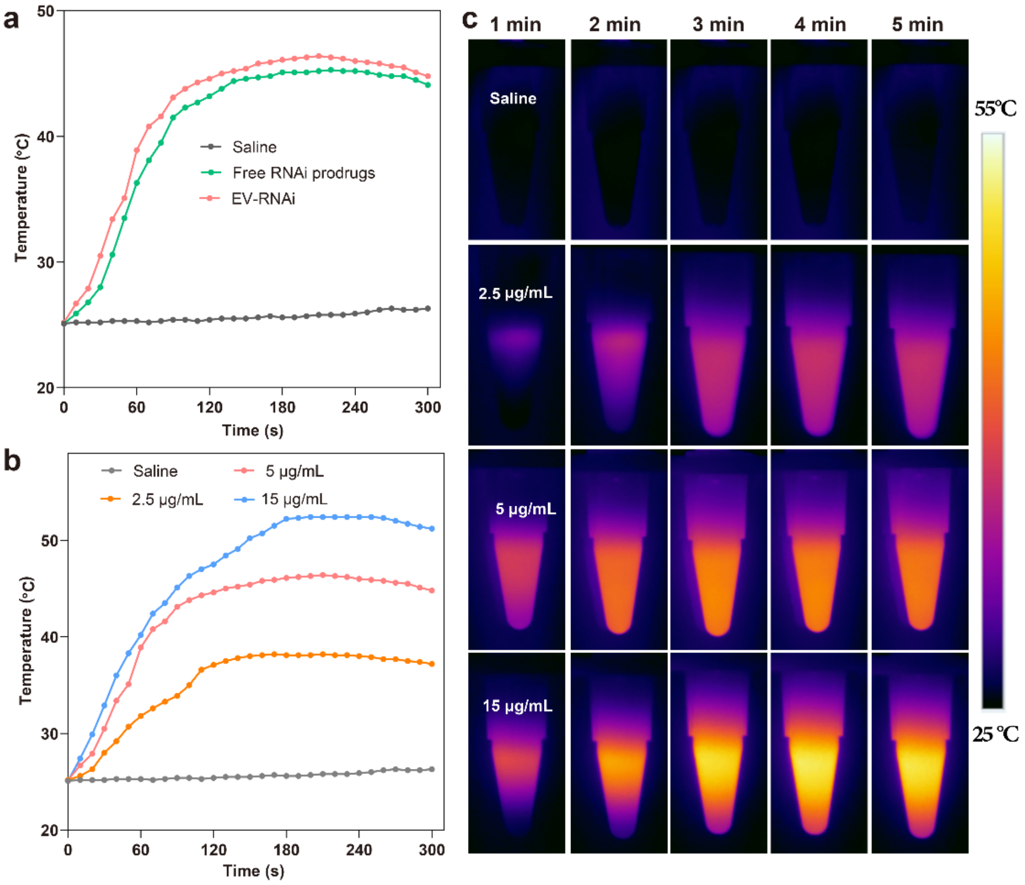

Supplementary Figure 28. Photothermal responses of ICG-labelled RNAi prodrugs. a, Photothermal conversion curve of saline, free RNAi prodrugs and RNAi prodrug-packaged EVs. **b,** Photothermal conversion curve of the different concentrations of RNAi prodrug-packaged EVs. **c,** The corresponding thermal images of different concentrations of RNAi prodrug-packaged EVs in Supplementary Figure 28b. The data are representative of three (**a**, **b**, **c**) independent experiments. All photoirradiation was carried out with an 808 nm laser (0.7 W/cm^2) for 5 min.

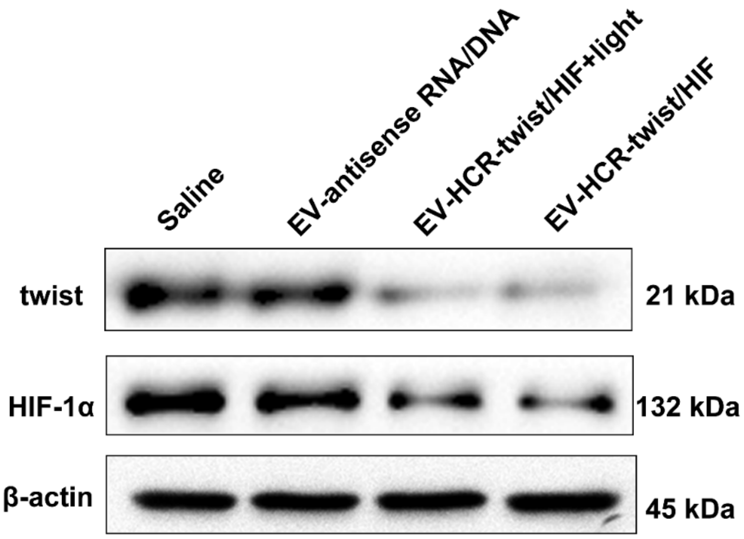

Supplementary Figure 29. Western blot analysis of the varied expressions of twist and HIF-1α protein in MDA-MB-231 cells with different treatments. Under hypoxia condition, MDA-MB-231 cells were treated with saline, EV-encapsulated antisense RNA/DNA hybrids, EV- encapsulated RNAi prodrugs with photoirradiation (0.7 W/cm^2, 5 min) or EV-encapsulated RNAi prodrugs for 36 h. The data are representative of three separate experiments.

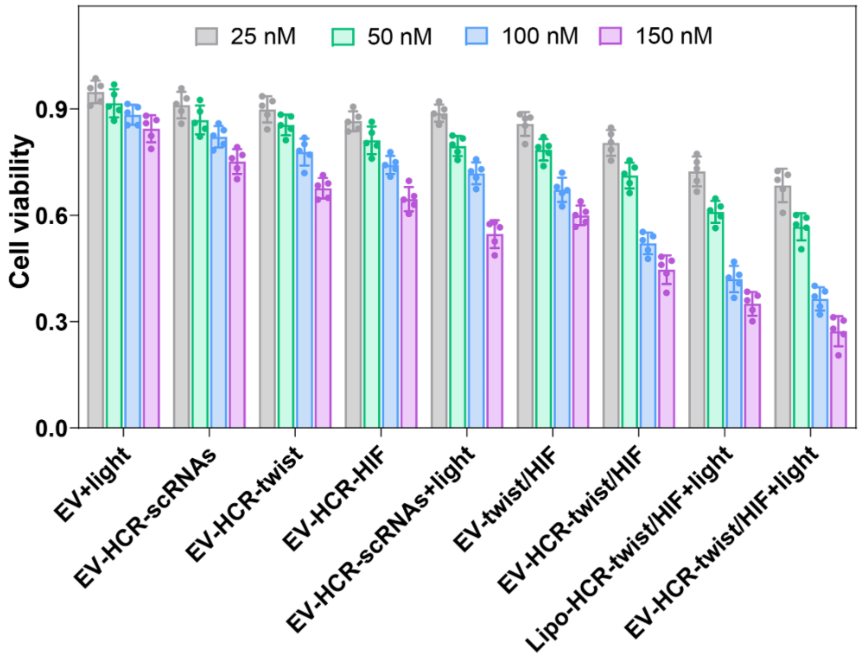

Supplementary Figure 30. Cell viability of the MDA-MB-231 cells after different treatments for 48 h under hypoxic condition. All photoirradiation was carried out with an 808 nm laser (0.7 W/cm^2) for 5 min. Data represent the mean ± s.d. of five independent replicates.

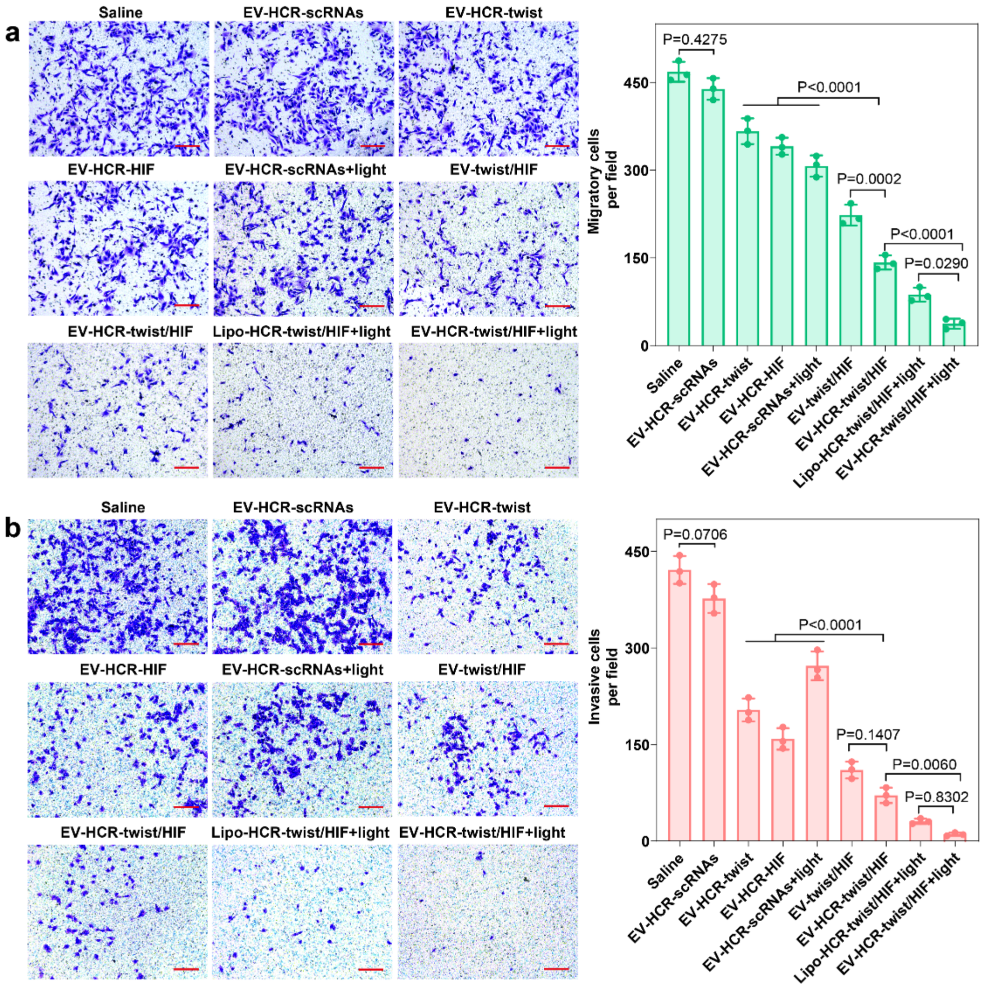

Supplementary Figure 31. **Cell motility and invasiveness analysis of differently treated MDA-MB-231 cells under hypoxia condition**. **a,**Transwell migration assay (left) and their corresponding quantification analysis (right) from differently treated MDA-MB-231 cells. **b,** Transwell Matrigel invasion assay (left) and their corresponding quantification analysis (right) from differently treated MDA-MB-231 cells. All photoirradiation was carried out with 808 nm laser (0.7 W/cm^2) for 5 min. All scale bars are 200 µm. Data represent the mean ± s.d. from three (**a**, **b**) independent replicates. Statistical significance (**a**, **b**) is calculated by two-way ANOVA followed by Bonferroni's multiple comparisons test.

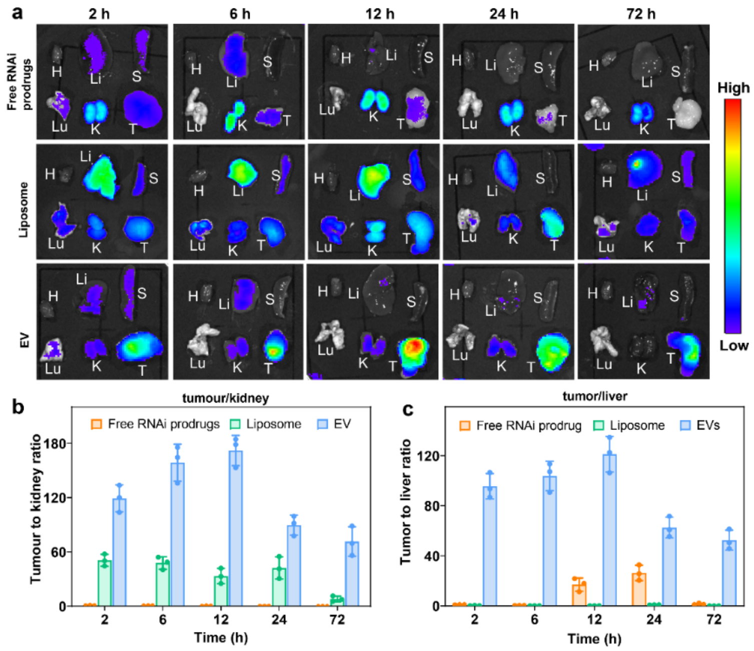

Supplementary Figure 32. Tissue distribution analyses. a, Ex vivo representative fluorescence images of the major organs after administration of free RNAi prodrugs, RNAi prodrug-loaded liposome (liposome) or RNAi prodrug-packaged EV (EV) to tumor-bearing mice at the indicated time points. H, heart; Li, Liver; S, spleen; Lu, lung; K, kidney; T, tumor. Quantitative analysis of the biodistribution in tumour to kidney **(b)** and tumour to liver ratio **(c)** at the indicated time points. Data represent the mean ± s.d. of three (**b**, **c**) independent replicates.

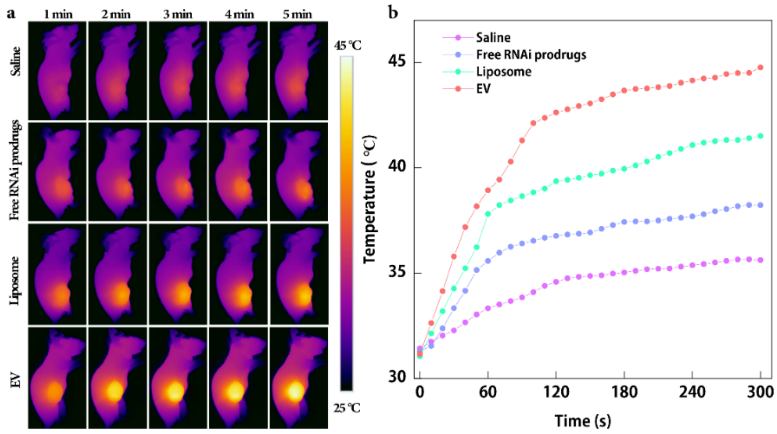

Supplementary Figure 33. **Photothermal characterization in vivo.** Representative thermal images **(a)** and the corresponding tumour temperature change **(b)** of mice at 12 h after intravenous injection of free RNAi prodrugs (1 mg), RNAi prodrug-loaded liposome (1 mg) or RNAi prodrug-packaged EV (0.5 mg) followed by an 808 nm photoirradiation (0.7 W/cm^2, 5min). Data are representative of three independent experiments.

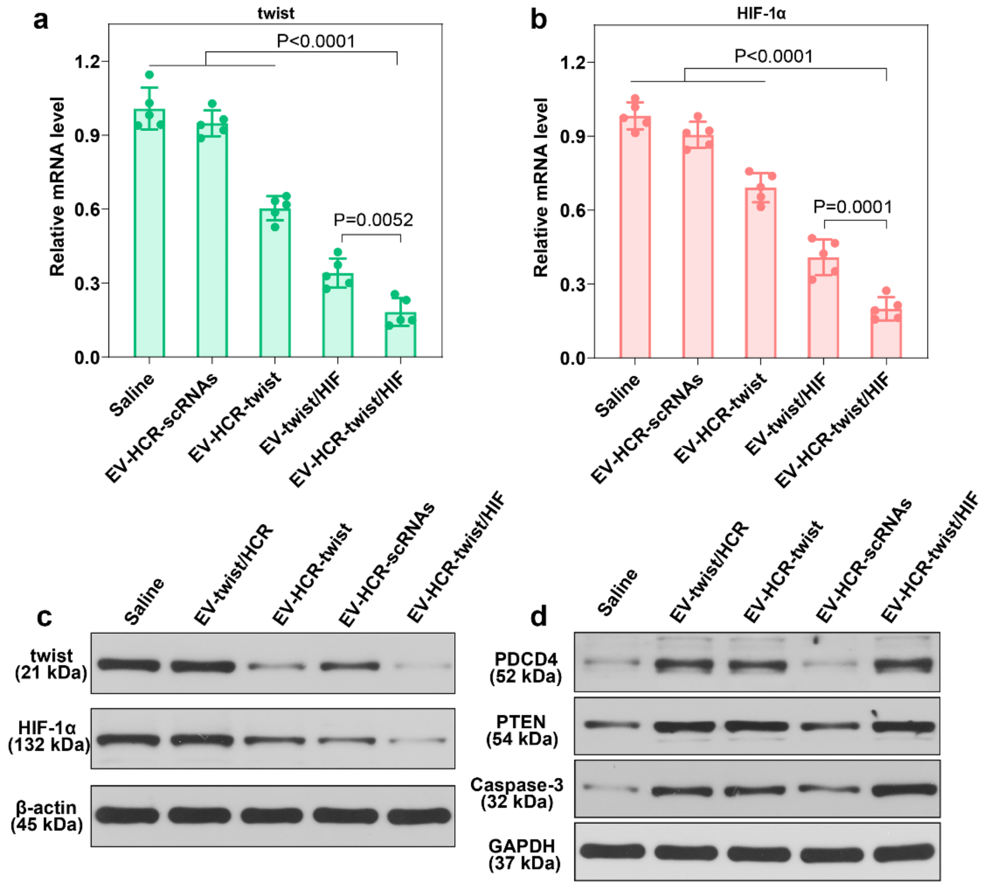

Supplementary Figure 34. The miR-21-activated gene silencing efficiency in tumours by intratumoral injection. (a, b) qRT-PCR analysis of twist and HIF-1α mRNA in MDA-MB-231 tumour-bearing mice after intravenous injected with different formulas on day five. **(c, d)** Western blot analysis of twist, HIF-1α, PDCD4, PTEN and Caspase-3 protein expression in MDA-MB-231 tumour-bearing mice after intravenous injected with different formulas on day five. Data represent the mean ± s.d. of five (**a**, **b**) independent replicates. The western blot results (**c**, **d**) are representative of three independent experiments. Statistical significance was calculated by one-way ANOVA followed by Tukey's post hoc test for (**a**) and (**b**).

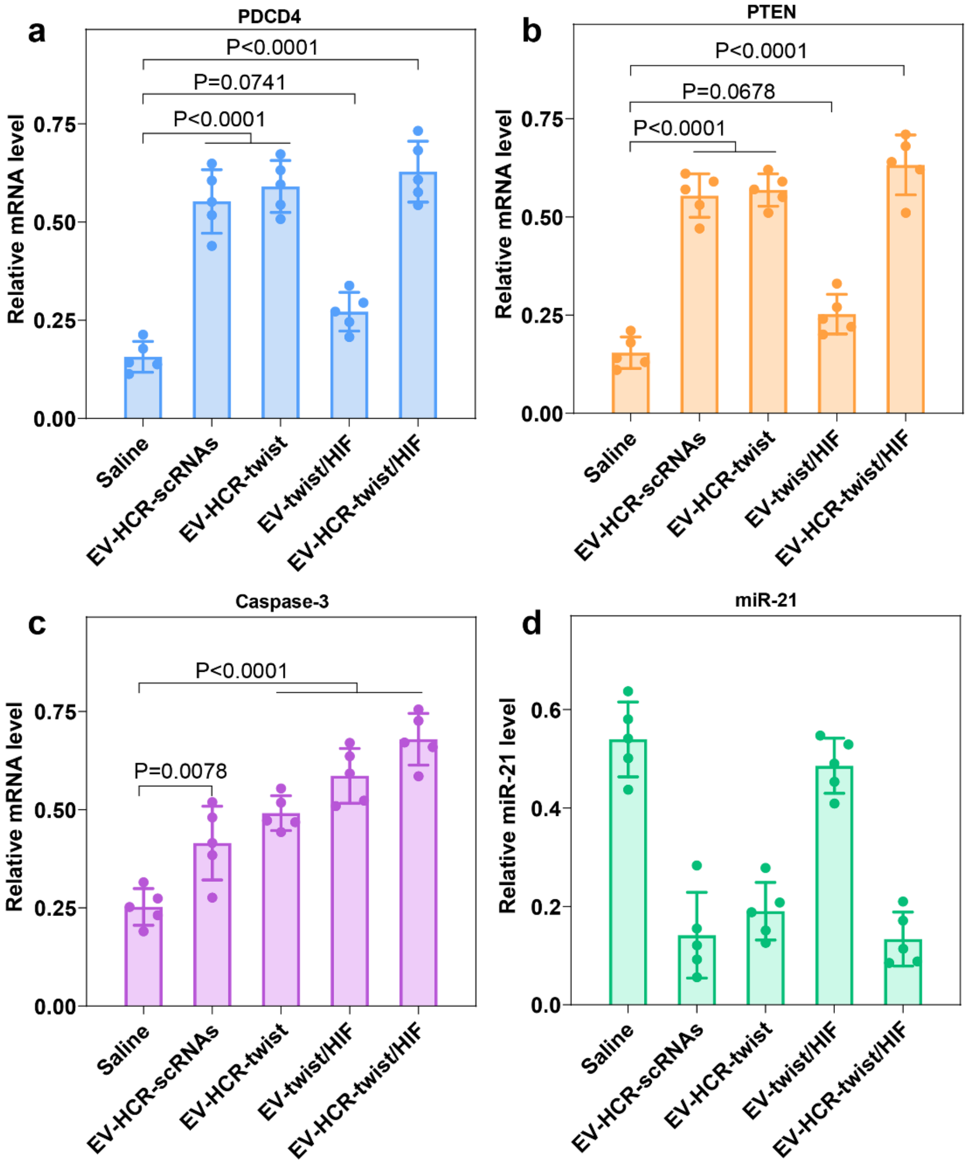

Supplementary Figure 35. **The miR-21-responsive bis-RNAi in tumours by intravenous administration.** qRT-PCR analysis of the expression of (a) PDCD4, (b) PTEN, (c) Caspase-3 and (d) miR-21 in mice tumour after intravenous injected with different formulas. Data represent the mean ± s.d. of five (**a-d**) independent replicates. Statistical significance was calculated by one-way ANOVA followed by Tukey's post hoc test for (**a**), (**b**) and (**c**).

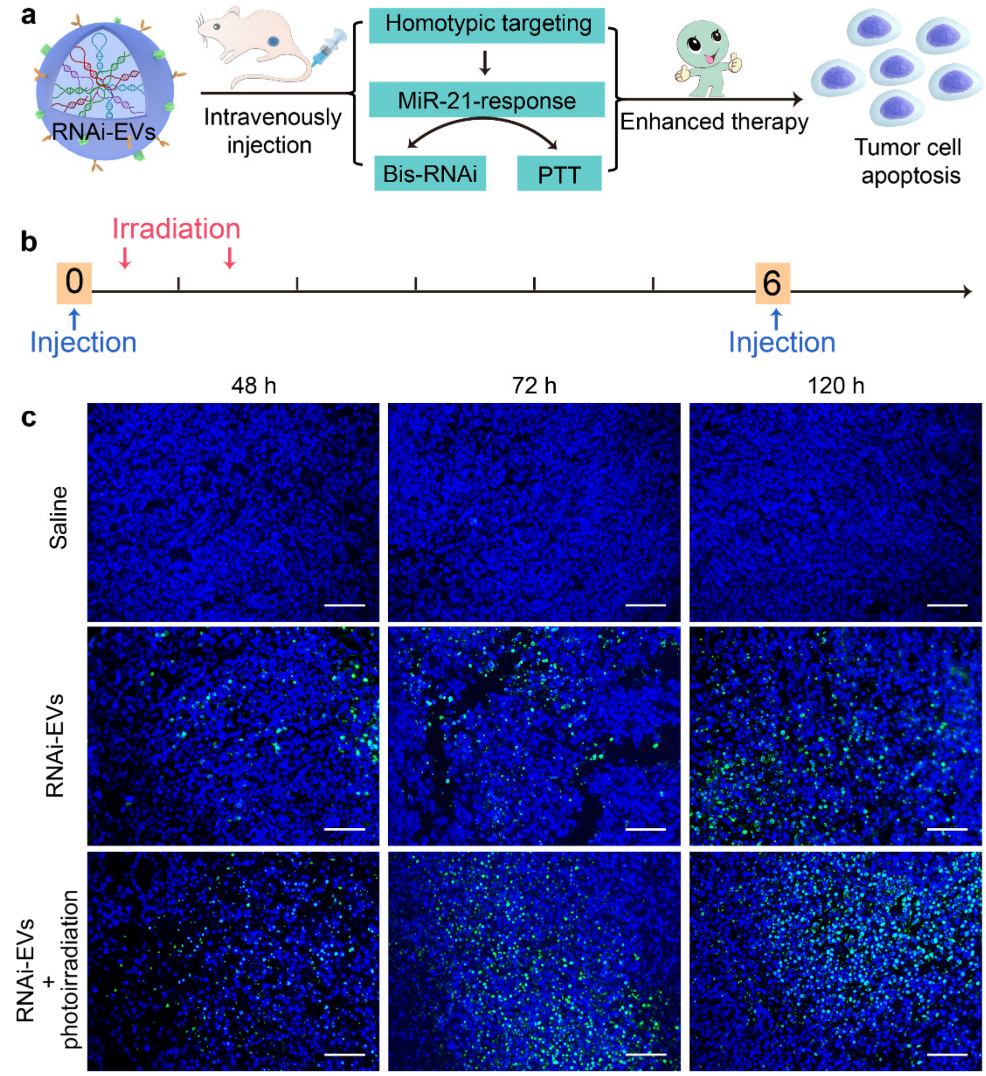

Supplementary Figure 36. Validation of the therapeutic performance of the first therapeutic procedure in vivo. a, Schematic illustration the Trojan EV-sustained cascade activation of multi-antenna gene silencing and auxiliary PTT. **b**, Therapeutic protocol of the administration of RNAi prodrugs in the subcutaneous MDA-MB-231 tumour model. **c**, Time-dependent TUNEL analysis of the corresponding MDA-MB-231 tumour tissues. The images are representative of three independent animals. All scale bars are 200 μm.

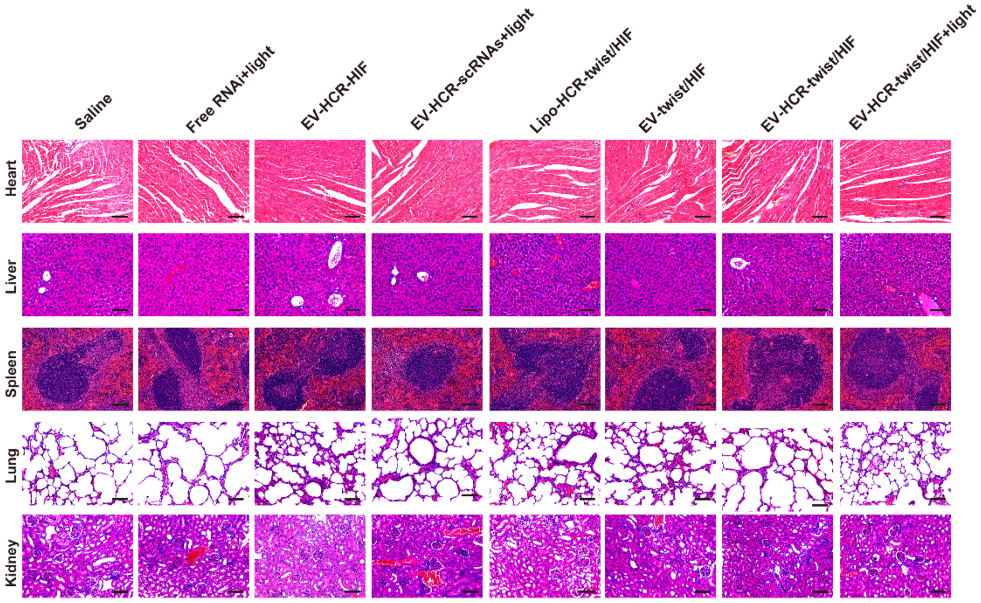

Supplementary Figure 37. Representative H&E-stained image of the main organs after these mice were sacrificed at 27 day post intravenous injection with different formulas. All photoirradiation was carried out with an 808 nm laser (0.7 W/cm^2) for 5 min. The images are representative of three independent animals. All scale bars are 200 μm.

Supplementary References

1. Woodrow, K. A., Cu, Y., Booth, C. J., Saucier-Sawyer, J. K., Wood, M. J., Saltzman, W. M. Intravaginal Gene Silencing Using Biodegradable Polymer Nanoparticles Densely Loaded With Small-Interfering RNA. *Nat. Mater*. **8**, 526-533 (2009).
2. Guo, R., Tian, Y., Yang, Y., Jiang, Q., Wang, Y., Yang, W. A Yolk-Shell Nanoplatform for Gene-Silencing-Enhanced Photolytic Ablation of Cancer. *Adv. Funct. Mater*. **28**, 1706398 (2018).
3. Lei, Y., Tang, L., Xie, Y., Xianyu, Y., Zhang, L., Wang, P., Hamada, Y., Jiang, K., Zheng, W., Jiang, X. Gold Nanoclusters Assisted Delivery of NGF siRNA for Effective Treatment of Pancreatic Cancer. *Nat. Commun*. **8**, 15130 (2017).
4. Bonoiu, A. C., Mahajan, S. D., Ding, H., Roy, I., Yong, K.-T., Kumar, R., Hu, R., Bergey, E. J., Schwartz, S. A., Prasad, P. N. Nanotechnology Approach for Drug Addiction Therapy: Gene Silencing Using Delivery of Gold Nanorod-siRNA Nanoplex in Dopaminergic Neurons. *Proc. Natl. Acad. Sci. U.S.A*. **106**, 5546-5550 (2009).
